# Lipidomic perspectives on the role of lactosylceramides in inflammation and disease: A narrative review

**DOI:** 10.1017/erm.2026.10054

**Published:** 2026-05-22

**Authors:** Dana Hicks, Luke Whiley

**Affiliations:** 1 Centre for Computational and Systems Medicine, Health Futures Institute, Murdoch University, Perth, WA, Australia; 2 School of Diagnostic & Therapeutic Science, Faculty of Health Sciences, Curtin University, Perth, WA, Australia; 3 Curtin Medical Research Institute (Curtin-MRI), Curtin University, Perth, WA, Australia; 4 Dementia Centre of Excellence, enAble Institute, Curtin University, Perth, WA, Australia

**Keywords:** cancer, cardiometabolic, glycosphingolipids immune, inflammation, lactosylceramide, lipidomics, neurodegeneration

## Abstract

**Background:**

Lactosylceramides (LacCers) are glycosphingolipids that play essential roles in physiological and pathological processes across immune, endocrine, and neurological systems, with mechanistic studies demonstrating that LacCers modulate inflammatory signalling, oxidative stress responses, membrane microdomain organisation, and control aspects of mitochondrial function. Historically, LacCers were quantified predominantly as a total lipid subclass, limiting the ability to discern how individual species contribute to biological processes in clinical contexts. Recent advances in mass spectrometry based lipidomics now enable LacCer species to be resolved by acyl-chain length and saturation, offering far greater biochemical and clinical insights.

**Methods:**

In this narrative review, we examine evidence from population based lipidomic studies describing how LacCer composition varies across healthy and diseased states.

**Results:**

In metabolic and vascular disorders, multiple studies report elevations in specific short- and medium-chain LacCer species, whereas patterns involving longer-chain species appear more heterogeneous. Altered LacCer profiles have also been described in neurodegenerative disease, chronic kidney disease, and cancers, with species-level differences varying by disease-context, tissue type, and analytical platform.

**Conclusions:**

Our findings describe disease- and tissue-specific variations in LacCer acyl-chain composition, underscoring the value of species-level resolution for mechanistic understanding and informing the application of LacCer profiles in future biomarker and therapeutic studies.

## Introduction

**Figure 1.**
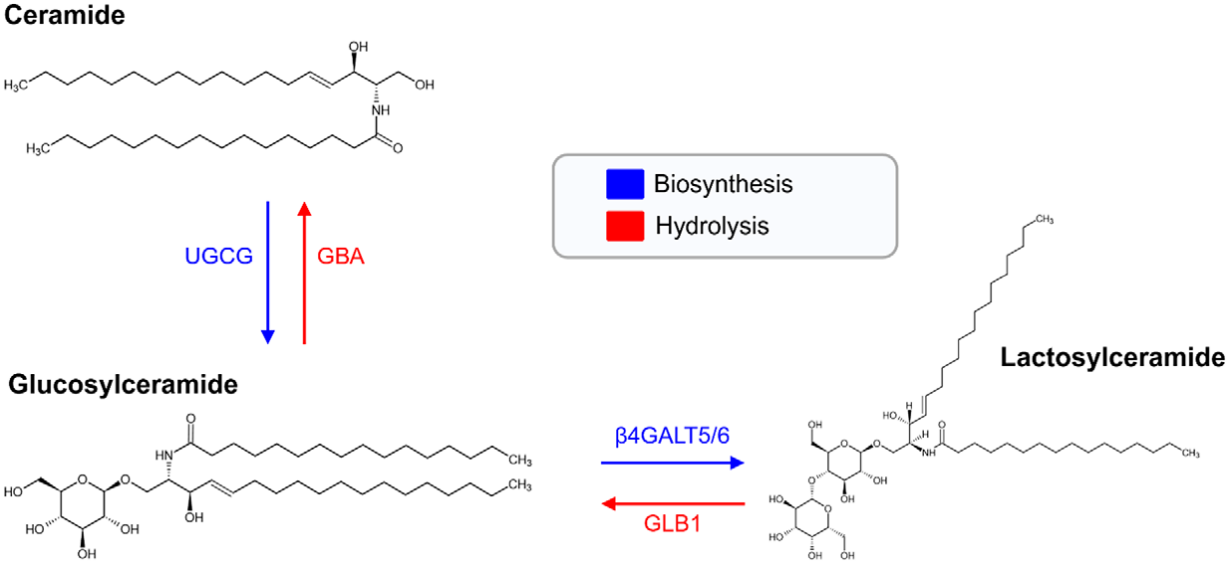
Lactosylceramide (LacCer) biosynthesis and lysosomal hydrolysis. Biosynthesis: Ceramide (Cer) is glycosylated by UDP-glucose:ceramide glucosyltransferase (UGCG), producing glucosylceramide (GlcCer). Beta-1,4-galactosyltransferase 5 (β4GALT5) or beta-1,4-galactosyltransferase 6 (β4GALT6) transfers a galactose moiety from UDP-galactose to GlcCer, forming lactosylceramide (LacCer). Hydrolysis: β-Galactosidase (GLB1) hydrolyses LacCer to GlcCer by removing the terminal galactose. β-Glucosidase (GBA) then cleaves GlcCer, yielding Cer. Figure 1. long description.

Lactosylceramides (LacCers) are a glycosphingolipid (GSL) composed of a ceramide backbone linked to a lactose moiety (Figure 1) (Ref. 1). They occupy a key branching point in sphingolipid metabolism and serve both structural and signalling roles (Ref. 2). The ceramide backbone exhibits acyl-chain length heterogeneity across C16-C24 species, which influences lipid microdomain structure (Ref. 3). As central intermediates in GSL metabolism, LacCers contribute to membrane organisation, cellular communication and the regulation of inflammatory pathways (Ref. 2). Emerging evidence suggests that host–microbiota interactions may influence sphingolipid metabolism, including ceramide pools (Ref. 4). LacCers are synthesised by the addition of galactose to glucosylceramide, a reaction catalysed by the lactosylceramide synthases β-1,4-galactosyltransferase 5 and 6 (β4GALT5 and β4GALT6) (Refs 1, 5) (Figure 1). These enzymes exist in both soluble and type II membrane-bound forms and are predominantly localised to the Golgi apparatus (Ref. 2). Beyond de novo synthesis LacCer homeostasis is maintained through lysosomal hydrolysis pathways that regenerate glucosylceramide and ceramide (Figure 1) (Ref. 6). Its expression is widespread, with high levels observed in the heart, brain, placenta, muscle and pancreas; lower levels in the lung and liver; and additional expression during fetal development in the brain, lung, liver and kidney (Ref. 7).

Within the total sphingolipid pool, LacCers account for about 5.8%, with LacCer(d18:1/16:0) being the most abundant species (Ref. 8). They are predominantly synthesised in the liver and delivered to extra-hepatic tissues, including the kidney on the surface monolayer of lipoproteins (Refs 1, 9, 10). It is distributed across very-low-density (VLDL), low-density (LDL) and high-density lipoprotein (HDL) fractions, making up approximately 3–13%, 36–57% and 34–54% of their respective total sphingolipid content (Refs 9, 10).

**Figure 2.**
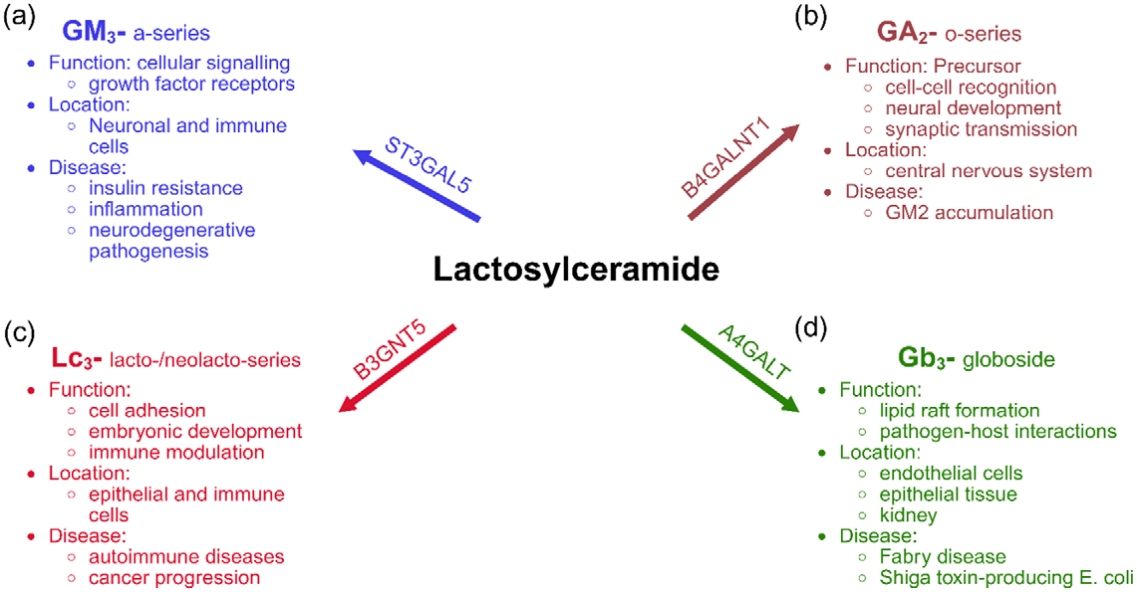
The four primary branches of glycosphingolipid (GSL) biosynthesis that initiate from the central intermediate Lactosylceramide (LacCer): (**A**) α-2,3-sialyltransferase (ST3GAL5) catalyses the formation of monosialodihexosylganglioside (GM_3_). GM_3_ can also be further sialylated by ST8SIA1 (GD3 synthase) to form GD3, the precursor of the b-series gangliosides; (**B**) β-1,4-N-acetylgalactosaminyltransferase (B4GALNT1) converts LacCers into asialo-GM_2_ (GA_2_); (**C**) β-1,3-N-acetylglucosaminyltransferase (B3GNT5) produces lactotriaosylceramide (Lc_3_), a precursor for the lacto- and neolacto-series GSLs involved in immune modulation and cancer progression and (**D)** α1–4-galactosyltransferase (A4GALT) synthesises globotriaosylceramide (Gb_3_). Figure 2. long description.

Downstream of its synthesis, LacCers function as central precursors for multiple GSL families that result from sequential addition of specific sugar residues with unique structural and functional properties (Figure 2). Monosialodihexosylganglioside (GM_3_) is the primary precursor for the a-series gangliosides (Figure 2A) and contributes to cellular signalling, including insulin receptor activity and neurodegenerative pathogenesis (Refs 11, 12). GM_3_ can also be further sialylated by ST8SIA1 (GD3 synthase) to form GD3, the precursor of the b-series gangliosides (Refs 12–14). Subsequent glycosylation generates disialylated gangliosides such as GD2, GD1b, GT1b and related structures (Refs 12–14). In parallel, LacCer derived o-series and lacto-neolacto-series GSL’s are involved in neural development, cell–cell recognition and specialised immune signalling, whilst globosides contribute to membrane organisation, lipid raft formation and implicated in pathogen–host interactions (Figure 2B–D) (Refs 11, 13). Collectively, these biosynthetic pathways highlight the pivotal role of LacCers in shaping membrane architecture, regulating intracellular processes, modulating immune function and contributing to diverse pathophysiological mechanisms.

For additional insight into LacCer biochemistry, readers are referred to the in-depth review by Chatterjee et al. (Ref. 2), which provides an in-depth overview of LacCer metabolism, transport and functional biology. Building on this foundation, our review takes a distinct approach by synthesising insights from systems lipidomic studies, with an emphasis on species-level changes that refine current understandings of LacCer function and responses in health and disease.

Initial studies that made biological measures of LacCer relied on thin-layer chromatography, immunoblotting and radiolabelled GSLs to quantify total LacCer in biopsy tissue, cell lines and animal models (Refs 15–20). These approaches provided evidence linking LacCer to chronic conditions such as cardiovascular disease (CVD), chronic inflammatory lung diseases, inflammatory bowel disease (IBD) and cancer (Refs 15–20) However, these approaches lacked the resolution to distinguish individual lipid species, masking the biological relevance of specific fatty acyl-chain variants. Lipidomic studies that use mass spectrometry (MS) have reported elevated total LacCer levels across various chronic inflammatory diseases, particularly those involving cardiometabolic dysfunction, e.g., atherosclerosis (Refs 21, 22), non-alcoholic steatohepatitis (NASH) (Ref. 23) and Type 2 Diabetes (T2D) (Refs 24, 25); immune-mediated injury, e.g., lupus nephritis (Ref. 26) and burn injury (Ref. 27); and neurodegeneration, e.g., Alzheimer’s disease (AD) (Refs 28, 29) and Parkinson’s disease (PD) (Ref. 29). While total LacCer is often elevated in these conditions, it has also been reported to decrease with biological ageing (Ref. 30), cancer metastasis (Ref. 31) and in T2D risk (Ref. 32). These conflicting trends of total LacCer levels in T2D likely reflect the cumulative effect of individual LacCer species, where chain-specific variations, some increasing and others decreasing, skew the overall trend at the LacCer subclass level.

Recent advances in liquid chromatography-tandem MS (LC–MS/MS) enable species-level identification and quantification of LacCers (Ref. 33). Therefore, this review builds upon the limitations of relying solely on total LacCer measurements and emphasises the need for detailed analyses to resolve the roles of individual LacCer species in specific biological contexts. Accordingly, all lipidomic studies reporting LacCer species included in this review are summarised in Supplementary Tables S1–S6 (Refs 34–80, 21–32), while key mechanistic findings are synthesised in the main text.

## Systems-level LacCer signatures and disease-associated alterations

LacCer is a bioactive GSL whose abundance has been associated with immune, metabolic and inflammatory responses across systemic contexts in health and disease. Lipidomic studies demonstrate that both the abundance and acyl-chain composition of LacCers vary across biological systems, including sex-specific differences, with emerging evidence also indicating variability across ethnic groups (Ref. 32). In the sections that follow, we synthesise these observations across the immune, endocrine, cardiovascular, central nervous and renal systems, and examine how LacCer dysregulation manifests in cancer. Together, these system-level signatures underscore the broad physiological relevance of LacCers and the complexity of its alterations across human systemic responses to disease.

### LacCers across immune and inflammatory system

LacCers are thought to play key roles in driving systemic and inflammatory responses, promoting recruitment of immune cells, cytokine signalling and endothelial activation across cardiometabolic, neural and tumour environments (Refs 1, 81, 82). Within cholesterol- and sphingolipid-rich lipid rafts, LacCers modulate nicotinamide adenine dinucleotide phosphate (NADPH) oxidase activity, triggering reactive oxygen species (ROS) production, adhesion molecule expression and pro-inflammatory cytokine release (Ref. 1).

Among LacCer species, unsaturated forms such as LacCer(d18:1/24:1) are particularly prone to oxidative modification (Ref. 83). These species undergo free-radical reactions that generate ROS and secondary products, contributing to endothelial dysfunction and facilitating the recruitment and activation of innate immune cells, particularly monocytes and neutrophils (Refs 35, 84). Consistent with these mechanisms, LacCer levels correlate positively with multiple inflammatory markers, including interleukin-6 (IL-6), monocyte chemoattractant protein-1 (MCP-1), macrophage inflammatory protein-1 beta (MIP-1β), C-reactive protein (CRP), fibrinogen and intercellular adhesion molecule 1 (ICAM-1), highlighting its role in systemic inflammation (Refs 36, 37). As a result, altered LacCer metabolism and composition have been observed in conditions that affect multiple organ systems.

LacCers are increasingly recognised as a central node in immune regulation, integrating innate immune signalling and adaptive immune responses through their role as a precursor of complex GSLs (Refs 13, 14, 84, 85). LacCers play several roles in innate immunity, functioning both as a structural component of neutrophil membranes and as a precursor to ganglioside GM_3_ (Ref. 14). Beyond its structural role, LacCer serves as a non-protein pattern-recognition receptor (PRR) that directly binds pathogen-associated molecular patterns (PAMPs), such as fungal β-glucans and bacterial lipoarabinomannan (Ref. 84).

Neutrophils, as key components of the innate immune system, drive the pathology of various inflammatory diseases through tissue infiltration and activation (Refs 86, 87). Their plasma membranes are enriched with specific LacCer species, namely (d18:1/24:0) and (d18:1/24:1), which are implicated in the formation of lipid rafts, cholesterol- and sphingolipid-rich microdomains, that facilitate signal transduction (Refs 3, 88). Within these rafts, LacCer interacts with αMβ2-integrin and the Src-family kinase Lyn, forming a signalling platform that enables receptor clustering and downstream activation (Ref. 84). Notably, αMβ2-integrin lacks intrinsic catalytic activity and instead relies on association with Lyn for intracellular signalling (Ref. 84). In neutrophils, these C24-containing LacCer species interact with the palmitoylated chains of Lyn within membrane rafts (Ref. 89). Upon pathogen binding, αMβ2-integrin translocates into Lyn-associated LacCer enriched rafts promoting outside-in signalling (Ref. 84). This is associated with NADPH oxidase activation and the subsequent production of ROS, contributing to neutrophil priming and effector function (Refs 84, 88).

Elevated concentrations of LacCer (d18:1/24:0) and (d18:1/24:1) have been identified in conditions characterised by increased neutrophil infiltration, including burn injury, where they discriminate non-severe burn injury from healthy paediatric individuals (Refs 27, 57). Increased concentrations have also been reported in the intima of atherosclerotic plaques (Ref. 39), in association with COVID-19 severity (Ref. 60) and in paediatric bacterial infections compared to viral infections (Ref. 58). These species are also elevated in lupus nephritis, a disease in which neutrophils contribute significantly to disease progression (Refs 26, 90). Collectively, these findings suggest that LacCer (d18:1/24:0) and (d18:1/24:1) may serve as biomarkers and functional mediators of neutrophil activity across multiple organ systems and inflammatory states.

Beyond neutrophil function, LacCer is the immediate precursor of the ganglioside GM_3_, with fatty-acyl chain composition largely retained upon sialylation by ST3GAL5 (Figure 2A) (Ref. 14). Under inflammatory conditions, ST3GAL5 expression and GM_3_ levels are dynamically regulated in a context-dependent manner, with several studies reporting increased GM_3_ synthesis in response to inflammatory and metabolic stress (Refs 12, 91). GM_3_ species act as acyl-chain-length–dependent modulators of Toll-like receptor 4 (TLR4) signalling, a key pathway in innate immune activation and metabolic inflammation (Ref. 14). Long-chain GM_3_ (LC-GM_3_) species, particularly GM_3_(d18:1/16:0), inhibit TLR4 activation, while very-LC-GM_3_ species, including GM_3_(d18:1/22:0) and GM_3_(d18:1/24:0), can enhance signalling if LC-GM_3_ species are depleted (Ref. 5). Due to the preservation of LacCer acyl-chain patterns in GM_3_, any alterations in LacCer species may mirror changes in GM_3_-mediated immune regulation (Ref. 14). Namely, increases in very-long-chain LacCers could indicate enhanced neutrophil priming, while depletion of LacCer(d18:1/16:0) may indicate GM_3_-driven activation of TLR4 signalling.

Alterations in LacCer acyl-chain composition are a recurring feature of immune-mediated inflammatory disorders, highlighting its potential role in disease pathogenesis. In respiratory infections, bacterial and viral aetiologies are associated with differing LacCer species and stage of disease dependency. For example, LacCer(d18:1/12:0) is increased in pneumonia versus upper respiratory tract infection (URI), and LacCer(d18:1/14:0) has been observed in recovered compared to acute pneumonia (Table 1) (Refs 58, 59). In digestive immune-mediated disorders, including paediatric ulcerative colitis (UC) and Crohn’s disease (CD), Salihovic et al. (Ref. 63) reported increased serum LacCer(d18:1/16:0) compared with symptomatic controls (Table 1). Similarly, Daniluk et al. (Ref. 61) showed that LacCer(d18:1/16:0) discriminated UC from CD in paediatric patients (Table 1), a finding further supported by Filimoniuk et al. (Ref. 62), who demonstrated positive correlations between LacCer(d18:1/16:0) and clinical inflammatory markers, including CRP, platelet count and white blood cell (WBC) count (Supplementary Table S2). In adults with UC, Bazarganipour et al. (Ref. 64) reported increased LacCer(d18:1/16:0) and LacCer(d18:1/24:0) in inflamed colonic tissue of UC participants, whereas serum LacCer(d18:1/24:1) was reduced in severe disease stages (Table 1). Collectively, these results suggest that the LacCer acyl-chain patterns are linked to distinct biological matrices, inflammatory states and immune pathways.

**Table 1.** Summary of altered lactosylceramide (LacCer) species between immune-mediated conditions Table 1. long description.

Condition	Matrix	LacCer (d18:1/12:0)	LacCer (d18:1/14:0)	LacCer (d18:1/16:0)	LacCer (d18:1/18:0)	LacCer (d18:1/18:1)	LacCer (d18:1/22:0)	LacCer (d18:1/24:0)	LacCer (d18:1/24:1)
Bacterial vs viral (Ref. 58)	Plasma Serum	n.r	n.r	↑	n.r	n.r	n.r	n.r	↑
Pneumonia vs URI (Ref. 59)	Serum	↑	n.s	n.r	n.r	n.r	n.r	n.r	n.r
Acute vs recovered pneumonia (Ref. 59)	Serum	n.s	↑	n.r	n.r	n.r	n.r	n.r	n.r
COVID–19 severity (Ref. 60)	Serum Urine	n.r	n.r	↑	n.r	n.r	↑	↑	n.s
UC (Ref. 64)	Colon tissue	n.r	n.r	↑	n.s	n.r	n.r	↑	n.s
Paediatric UC vs CD (Ref. 61)	Serum	n.r	n.r	↑	n.r	n.r	n.r	n.r	n.r
Paediatric UC vs CD (Ref. 62)	Serum	n.r	n.r	↑	n.s	n.s	n.s	n.s	n.r
Paediatric UC/CD * (Ref. 63)	Plasma Serum	n.r	n.r	↑	n.r	n.r	n.r	n.r	n.r

*Note:* ↑: increased vs comparison; ↓: decreased vs comparison; *: condition vs control; CD: Crohn’s disease; n.r: not reported; n.s: no significance reported; UC: ulcerative colitis; URI: upper respiratory tract infection.

### Endocrine system

Mechanistically, LacCers influence insulin signalling indirectly by converting to GM_3_ (Figure 2A), an established modulator of insulin receptor function (Refs 12, 92). GM_3_ accumulation alters the structure of lipid rafts in insulin-sensitive tissues, including the liver, skeletal muscle and adipose tissue (Refs 92, 93). This impairs insulin receptor phosphorylation and reduces downstream signalling through insulin receptor substrate-1 (IRS-1) and the phosphoinositide 3-kinase (PI3K)/protein kinase B (Akt) pathway (Refs 92, 93). As a result, glucose transporter type 4 (GLUT-4) translocation is diminished, which limits glucose uptake and promotes insulin resistance (IR) (Refs 92, 93). This is likely compounded by LacCer, which induces the activation of NADPH oxidase within lipid rafts, promoting ROS generation and inflammatory signalling, thereby amplifying IR and endothelial dysfunction (Ref. 1).

TLR4 signalling is increasingly recognised as a mediator of obesity and metabolic disorders, linking innate immune activation to endocrine dysfunction (Ref. 14). Among the studies examining LacCer species in metabolic states, most focused on associations with insulin resistance, glucose homeostasis and vascular function (Refs 24, 32, 37, 41–49) (Supplementary Table S2). Notably, one study in metabolic syndrome reported that total LacCer levels were significantly associated with increased inflammatory markers, including IL-6, CRP, fibrinogen and ICAM, which are well-established downstream markers of TLR4/NF-κB signalling (Ref. 37). Taken together, these findings suggest that LacCer variation in endocrine disorders may influence metabolic inflammation through GM_3_-mediated regulation of TLR4 signalling, although further studies are needed to confirm this mechanism.

Individual LacCer species appear to track across the differing metabolic phenotypes. In contrast, metabolic syndrome (MetS) and IR are inversely associated with LacCer(d18:1/14:0), (d18:1/16:0) and (d18:1/24:1), alongside both HOMA-IR and T2D (Table 2) (Refs 37, 45). However, LacCer(d18:1/18:1) exhibits a positive association with HOMA-IR (Supplementary Table S2), suggesting that the metabolic burden of LacCer is driven by specific acyl-chain remodelling rather than a uniform shift across all species (Ref. 37). Notably, while these specific species are reduced, total LacCer levels remain positively associated with systemic inflammatory markers, such as IL-6 and CRP (Supplementary Table S2) (Ref. 37).

**Table 2.** Summary of altered lactosylceramide (LacCer) species between endocrine-mediated conditions Table 2. long description.

Condition	Matrix	LacCer (d18:1/12:0)	LacCer (d18:1/14:0)	LacCer (d18:1/16:0)	LacCer (d18:1/18:0)	LacCer (d18:1/18:1)	LacCer (d18:1/20:0)	LacCer (d18:1/22:0)	LacCer (d18:1/24:0)	LacCer (d18:1/24:1)	LacCer (d18:1/26:0)
Overweight (Ref. 41)	Serum	↑	n.r	n.r	n.r	n.r	n.r	n.r	n.r	n.r	n.r
MetS (Ref. 37)	Serum	n.r	↓	↓	n.s	n.s	n.r	n.s	n.s	↓	n.r
Obesity ± MetS (Ref. 42)	Plasma	n.r	n.r	n.s	n.s	n.s	n.s	n.s	n.s	n.s	n.r
IFG (Ref. 43)	Serum	↑	n.r	n.r	n.r	n.r	n.r	n.r	n.r	n.r	n.r
IFG (Ref. 44)	Plasma	n.r	n.r	↓	n.r	n.r	n.r	n.r	n.r	n.r	n.r
IR + T2D (Ref. 45)	Plasma Serum	n.r	↓	↓	n.s	n.s	n.r	n.s	n.s	↓	n.r
IR in obesity ± T2D (Ref. 46)	Serum	n.r	n.s	n.s	n.s	n.r	n.s	↓	n.s	n.s	n.r
T2D risk (Ref. 47)	Plasma	n.r	↓	↓	n.s	n.s	↓	↓	n.s	↓	n.r
T2D risk (Ref. 48)	Plasma	n.r	n.r	n.s	n.r	n.r	n.r	n.r	n.r	n.r	n.r
T2D risk (Ref. 32)	Plasma	n.r	n.r	n.r	n.r	↓	n.r	n.r	n.r	n.r	n.r
Condition	Matrix	LacCer (d18:1/12:0)	LacCer (d18:1/14:0)	LacCer (d18:1/16:0)	LacCer (d18:1/18:0)	LacCer (d18:1/18:1)	LacCer (d18:1/20:0)	LacCer (d18:1/22:0)	LacCer (d18:1/24:0)	LacCer (d18:1/24:1)	LacCer (d18:1/26:0)
Obesity ± T2D (Ref. 49)	Adipo-somes	n.r	n.r	↑	↑	n.r	↑	n.r	n.r	↑	n.r
T2D (Ref. 24)	Plasma	n.r	n.r	n.r	↑	n.r	↑	n.s	↑	n.s	↑
T2D + MA (Ref. 50)	Plasma	n.r	n.r	n.r	↓	n.r	↓	↑	↓	↑	↓
NAFLD (Ref. 23)	Serum	n.r	↑	n.s	n.s	n.r	n.s	n.s	n.s	n.s	n.r
NASH (Ref. 23)	Liver	n.r	n.s	n.s	n.s	n.r	n.s	n.s	n.s	↑	n.r
NAFLD (Ref. 52)	Serum	n.r	n.r	↓	n.r	n.r	n.r	n.r	n.r	n.r	n.r
NAFLD (Ref. 53)	Serum	n.r	n.r	↓	n.r	n.r	n.r	n.r	n.r	↓	n.r
PCOS ± obesity ± IR (Ref. 56)	Serum	n.s	n.r	↓	n.s	n.r	n.s	n.s	n.s	n.s	n.r

*Note:* ↑: increased vs control; ↓: decreased vs control; IFG: impaired fasting glucose; IR: insulin resistance; MA: macroalbuminuria; MetS: metabolic syndrome; NAFLD: non-alcoholic fatty liver disease; NASH: non-alcoholic steatohepatitis; n.r: not reported; n.s: no significance reported; PCOS: polycystic ovarian syndrome; T2D: type 2 diabetes mellitus.

In the serum of obese (BMI 28.0–39.9 kg/m^2^) individuals with IR, LacCer(d18:1/14:0) associates positively with intramuscular triglyceride accumulation (Supplementary Table S2), whereas LacCer(d18:1/22:0) is predictive of whole-body IR (Table 2) (Ref. 46). In contrast, plasma studies of T2D risk show divergent trends. In women with gestational diabetes, LacCer species, including (d18:1/14:0), (d18:1/16:0), (d18:1/20:0), (d18:1/22:0) and (d18:1/24:1) (Table 2), are negatively associated with the 8-year progression to T2D (Ref. 47). Furthermore, total plasma LacCer, along with the (d18:1/18:1) and (d18:1/18:2) species, was found to be negatively associated with T2D risk in a separate study (Table 2) (Ref. 32). Beyond the systemic circulation, LacCer levels were significantly altered within adiposomes from obese participants (BMI > 30.0 kg/m^2^) with T2D (Ref. 49). Adiposomes from obese patients with T2D exhibit enriched LacCer(d18:1/16:0), (d18:1/18:0), (d18:1/20:0) and (d18:2/24:1) (Table 2), with the authors reporting that the uptake and fusion of these adiposomes with endothelial cells was fourfold higher than in controls, leading to an increase of LacCer(d18:1/16:0) and (d18:1/18:0) in these cells (Ref. 49). These findings suggest that whilst plasma LacCer levels may vary with T2D risk, these specific species are sequestered and transported via adiposomes, which may contribute to endothelial dysfunction.

The association between LacCers and metabolic dysfunction extends to hepatic lipid accumulation, where findings vary according to both the tissue compartment and the degree of underlying pathology. In serum, LacCer(d18:1/14:0) is positively associated with the presence of non-alcoholic fatty liver disease (NAFLD) (Table 2) (Ref. 23). In liver tissue, however, both total LacCer and the (d18:1/24:1) species are increased in patients with non-alcoholic steatohepatitis (NASH) (Table 2) (Ref. 23). Other serum analyses indicate that LacCer(d18:1/16:0) is associated with higher hepatic attenuation, despite exhibiting an inverse association with fasting glucose and T2D incidence over 5 years (Ref. 52). In contrast, LacCer(d18:1/16:0) and (d18:1/24:1) in both serum and liver tissue have been associated with lower hepatic triglyceride levels (Ref. 53). Further shifts are evident in visceral adipose tissue, where LacCer(d18:1/14:0), (d18:1/16:0), (d18:1/24:0) and (d18:1/24:1) exhibit positive associations with adiponectin (Ref. 23).

Collectively, these findings indicate that LacCer variation across metabolic disorders is attributed to specific acyl-chain reorganisation and its distribution across differing tissue types. Whether these variations are driven by inflammatory or metabolic signalling remains to be determined, highlighting an important gap in our mechanistic understanding of endocrine dysfunction.

### Cardiometabolic health

LacCers have been implicated as a mediator of vascular inflammation and endothelial dysfunction, processes central to atherosclerosis development and progression. Mechanistically, LacCer activates protein kinase C alpha and epsilon isoforms (PKCα/ε) and phospholipase A₂ pathways in monocytes (Ref. 94). This activation leads to upregulation of platelet endothelial cell adhesion molecule-1 (PECAM-1) and increases monocyte adhesion to endothelial cells (Ref. 94). These events promote vascular inflammation and contribute to early atherogenic processes.

LacCer(d18:1/12:0) is elevated in overweight individuals (BMI 25–<30 kg/m^2^) compared with normal-weight individuals (BMI 18.5–<25 kg/m^2^) and positively correlates with brachial–ankle pulse wave velocity (baPWV), a marker of arterial stiffness and cardiovascular risk (Ref. 41). Elevations in LacCer(d18:1/12:0) characterise both overweight individuals (BMI 25.0–29.9 kg/m^2^) and those with impaired fasting glucose (IFG) (Table 2), where it further correlates with arterial stiffness (Supplementary Table S2) (Refs 41, 43). In a separate study, LacCer(d18:1/12:0) was positively associated with both fasting glucose and baPWV, supporting a mechanistic link between arterial stiffness and cardiovascular risk (Ref. 43). Chatterjee et al. (Ref. 39) examined LacCer distribution across arterial layers, categorising intima samples as normal (no lesions), fibrous plaques without calcification, or plaques with calcification; in each case, the intima was separated from the media before analysis. LacCer(d18:1/16:0), (d18:1/22:0), (d18:1/22:1) and (d18:1/24:0) were enriched in the intima of atherosclerotic plaques compared with unaffected aortic intima, whereas LacCer(d18:1/18:0) and (d18:1/18:1) were reduced, and LacCer(d18:1/16:1), (d18:1/17:0), (d18:1/18:2) and (d18:1/20:1) were absent (Ref. 39). In systemic lupus erythematosus (SLE), circulating LacCer species show distinct vascular associations. LacCer(d18:1/24:1) negatively correlated with total plaque area (TPA) at baseline but positively correlated with TPA after one year of follow-up, while LacCer(d18:1/26:0) and (d18:1/26:1) differentiated individuals with atherosclerosis from those without (Refs 22, 34). Notably, LacCer(d18:1/16:0) and (d18:1/24:0) were enriched in aortic plaques but inversely associated with age- and sex-adjusted TPA in SLE (Refs 22, 34). This contrast between tissue enrichment and plasma associations may reflect distinct local versus systemic regulation of LacCer species or immune-mediated mechanisms specific to SLE. Significantly, LacCer(d18:1/16:0) and (d18:1/24:0), while enriched in aortic plaques, were inversely associated with age- and sex-adjusted TPA in a SLE cohort (Refs 22, 39). This contrast between arterial enrichment and plasma associations may reflect distinct local versus systemic LacCer species regulation or immune-mediated mechanisms specific to SLE.

These findings suggest that LacCers may contribute to atherogenesis through processes shaped by their localisation in the arterial wall and inflammatory influences. However, whether these inflammatory influences are local, systemic, or both remains unknown, highlighting an important gap in our understanding of how LacCers contribute to atherosclerosis.

### Central nervous system

The brain is particularly vulnerable to disruptions in lipid metabolism due to its exceptionally high lipid content and reliance on GSLs, including LacCers, for membrane structure, signalling and synaptic function (Refs 11, 95). Mechanistically, LacCer is a pivotal intermediate in both ceramide and broader GSL metabolism (Figures 1 and 2). In addition to synthesis from ceramide, LacCers can also arise through ganglioside remodelling. For example, binding of elastin-derived peptides to the elastin receptor complex leads to activation of the neuraminidase-1 (NEU1) sialidase subunit, which converts GM_3_ to LacCers, leading to extracellular signal-regulated kinase (ERK) activation (Ref. 97). This NEU1-dependent route provides an additional mechanism by which altered LacCer metabolism may contribute to neurodegenerative diseases.

The accumulation of LacCers contributes to the disruption of plasma membrane microdomains, particularly lipid rafts, thereby impairing neuronal signalling and promoting the generation of ROS, which impairs mitochondrial function (Refs 1, 2). Within neurons, astrocytes and microglia, LacCer-enriched lipid rafts facilitate inflammation that exacerbates oxidative damage in the brain (Refs 82, 98, 99). In astrocytes, LacCer activates nuclear factor-κB (NF-κB), promoting chemokine release, such as MCP-1 (Refs 82, 99). Under these pro-inflammatory conditions, the accumulation of LacCers result in the dysregulation of GSL hydrolases and elevated cholesterol in lipid rafts (Ref. 12). The resulting activation of mitogen-activated protein kinase (MAPK), ERK 1/2 and NF-κB signalling pathways leads to astrocyte proliferation and neuronal apoptosis (Ref. 82). Additionally, LacCer accumulation dysregulates ganglioside metabolism by enriching GM_3_, which compromises membrane stability, reduces lipid raft fluidity and impairs signal transduction (Figure 2A) (Refs 12, 82). Altered GSL composition in lysosomes and plasma membranes exacerbates neurodegenerative processes (Refs 12, 82).

Beyond its role in catabolism, NEU1 participates in receptor signalling within lipid rafts, activating ERK1/2 and NF-κB pathways and is implicated in Alzheimer’s disease (AD) pathology (Refs 96, 100). Overall, dysregulated LacCer metabolism is linked to oxidative stress, neuroinflammatory signalling, compromised synaptic function and progressive neuronal loss across a range of neurodegenerative conditions (Refs 82, 83). However, these disorders differ markedly in their underlying molecular drivers, including protein aggregation, immune-mediated injury and cell-type specificity, which provides a framework for interpreting disease-specific LacCer signatures.

Neurodegenerative disorders are classified by their dominant pathogenic protein aggregates; however, many conditions exhibit overlapping or comorbid pathologies (Refs 29, 104–107). α-Synucleinopathies, including idiopathic Parkinson’s disease (IPD), dementia with Lewy bodies (DLB), multiple system atrophy (MSA) and GBA1-associated Parkinson’s disease (GBA1-PD), are defined by α-synuclein accumulation, yet may coexist with additional aggregates such as tau or amyloid-β, particularly in DLB and advanced disease stages (Refs 29, 81, 101–104). AD is characterised by amyloid-β plaques and tau neurofibrillary tangles, while progressive supranuclear palsy (PSP) is a primary tauopathy (Ref. 29). In contrast, multiple sclerosis is a chronic immune-mediated disorder defined by multifocal CNS inflammation, demyelination and gliosis rather than protein aggregation (Ref. 108). Distinct LacCer profiles appear to align with the primary protein aggregates across neurodegenerative disorders. Disorders dominated by α-synuclein pathology (IPD, DLB, GBA1-PD and MSA) showed increases in LacCer (d18:1/14:0), (d18:1/16:0) and (d18:1/16:1) (Table 3) (Ref. 29). DLB further includes the unsaturated species (d18:1/20:1), (d18:1/22:1), (d18:1/22:2), (d18:1/24:1) and (d18:1/24:2) (Table 3) (Ref. 29). AD results also include increases of LacCer(d18:1/14:0) and (d18:1/16:0) but additionally includes unsaturated species LacCer (d18:1/20:1), (d18:1/22:1) and (d18:1/22:2), suggesting further remodelling linked to mixed pathology (Table 3) (Ref. 29). However, PSP contrasted with the broader changes observed in AD (Table 3) (Ref. 29). Immune-mediated disorders show a distinct signature with RRMS characterised by increases in LacCer (d18:1/22:0) and reductions in (d18:1/20:1) (Table 3) (Ref. 72). LacCer (d18:1/16:0), (d18:1/16:1), (d18:1/22:0) and (d18:1/24:1) are increased in PMS, alongside the same reduction in LacCer (d18:1/20:1) (Table 3) (Ref. 72). Overall, these results suggest that LacCer (d18:1/14:0) and (d18:1/16:0) are common across the proteinopathies (Table 3). In contrast, distinct unsaturated and very-long LacCer species help distinguish AD and DLB from α-synuclein disorders, and immune-driven processes define a unique LacCer profile. However, the in-depth interpretation remains limited by data availability, underscoring the need for additional studies to clarify the mechanistic links between LacCer metabolism and neurodegenerative pathology.

**Table 3.** Summary of altered lactosylceramide (LacCer) species between neurodegenerative disorders Table 3. long description.

LacCer species	IPD (Ref. 29)	DLB (Ref. 29)	AD (Ref. 29)	PSP (Ref. 29)	MSA (Ref. 29)	*GBA1*-PD (plasma) (Ref. 75)	*GBA1*-PD (CSF) (Ref. 75)	RRMS (Ref. 72)	PMS (Ref. 72)
d18:1/14:0	↑	↑	↑	↑	↑	n.r	n.r	n.r	n.r
d18:1/16:0	↑	↑	↑	↑	↑	n.s	n.s	n.s	↑
d18:1/16:1	↑	↑	n.s	↑	↑	n.r	n.r	n.s	↑
d18:1/18:0	n.s	↑	↑	n.s	↑	↑	↓	n.r	n.r
d18:1/18:1	n.s	↑	n.s	n.s	n.s	n.r	n.r	n.s	n.s
d18:1/20:0	n.s	n.s	n.s	n.s	n.s	↓	↓	n.s	n.s
d18:1/20:1	n.s	↑	↑	n.s	n.s	n.r	n.r	↓	↓
d18:1/22:0	n.s	n.s	n.s	n.s	n.s	↑	n.s	↑	↑
d18:1/22:1	n.s	↑	↑	n.s	n.s	↑	n.s	n.s	n.s
d18:1/22:2	n.s	↑	↑	n.s	n.s	n.r	n.r	n.r	n.r
d18:1/24:0	n.s	n.s	n.s	n.s	↓	n.s	n.s	n.s	n.s
d18:1/24:1	n.s	↑	n.s	n.s	n.s	n.s	n.s	n.s	↑
d18:1/24:2	n.s	↑	n.s	n.s	n.s	n.r	n.r	n.r	n.r

*Note*: ↑: increased vs controls; ↓: decreased vs controls; AD: Alzheimer’s disease; CSF: cerebrospinal fluid; DLB: dementia with Lewy bodies; GBA-PD: GBA1-associated Parkinson’s disease; IPD: idiopathic Parkinson’s disease; LacCer: lactosylceramide; MSA: multiple system atrophy; n.r: not reported; n.s: no significance reported; PMS: progressive multiple sclerosis; PSP: progressive supranuclear palsy; RRMS: relapsing–remitting multiple sclerosis.

### Renal system

LacCers function as a mediator of renal signalling and disease progression. Under physiological conditions, LacCer synthesis is restrained by a low-density lipoprotein receptor (LDLR)-dependent pathway, in which native LDL exerts a dose-dependent inhibition (Refs 1, 16, 109). This regulatory mechanism is disrupted under conditions of oxidative stress, where methylation of lysine residues impairs LDLR recognition and weakens feedback control of LacCer synthesis (Ref. 109). Consistent with impaired LDLR-mediated regulation in human disease, multiple LacCer species, including LacCer(d18:1/16:0), (d18:1/18:0), (d18:1/20:0), (d18:1/22:0), (d18:1/24:0) and (d18:1/24:1), are increased within the LDL fraction in individuals with CKD compared with controls (Supplementary Table S5), indicating altered LacCer trafficking within circulating lipoproteins (Ref. 51). As a result, LDL uptake is redirected towards scavenger receptor pathways, leading to increased LacCers being generated and subsequent activation of ERK1/2 (p44 MAPK) signalling (Refs 1, 16, 78, 110). Experimental models indicate that NEU1 activity rises prior to LacCer accumulation, implicating GSL remodelling as an early trigger in renal inflammation (Ref. 77). Consistent with this disease-associated shift, basal p44 MAPK activity has been reported to be elevated in proximal tubular cells from those with autosomal dominant polycystic kidney disease (ADPKD), compared to controls (Ref. 78). This was built upon previous research by Chatterjee et al. (Ref. 111), who observed increased epidermal growth factor (EGF) activity and LacCer synthesis, supporting the dysregulation of LacCer-linked signalling pathways in renal disease.

Renal disease is associated with alterations in LacCer profiles across plasma, serum, urine and kidney tissue, indicating that LacCer remodelling accompanies kidney injury irrespective of the initiating condition. Lopes-Virella et al. (Ref. 112) reported that LacCer profiles differ by kidney disease stage, with microalbuminuria (MA) and CKD showing distinct patterns in plasma (Supplementary Table S5). In MA, circulating short-chain species LacCer(d18:1/14:0), (d18:1/16:0) and (d18:1/18:0) were reduced, while LacCer(d18:1/22:1) was increased (Supplementary Table S5) (Ref. 112). With progression to CKD, depletion of the short-chain species persisted, and additional very-long-chain species such as LacCer(d18:1/24:0) and (d18:1/26:1) (Supplementary Table S5) also decreased, reflecting a stage-dependent remodelling of LacCers rather than uniform loss (Ref. 112). The progressive rise in LacCer (d18:1/22:1) from control to MA, CKD and combined MA + CKD suggests that it tracks renal injury and may serve as a marker of disease progression (Ref. 112). Similar LacCer alterations were observed in paediatric CKD, with serum LacCer(d18:1/16:0) and (d18:1/24:0) elevated relative to controls (Supplementary Table S5). However, the opposing direction of these species compared with the aforementioned adult CKD cohort may reflect potential age or matrix differences in LacCer metabolism (Ref. 76). Urinary LacCer(d18:1/16:0) was elevated in SLE nephritis patients (Supplementary Table S5), whereas plasma levels remained unchanged, indicating that urine may be a more sensitive matrix for detecting renal LacCer perturbations (Ref. 77). In lupus nephritis, Wolf et al. (Ref. 26) expanded upon these findings by demonstrating broader increases in LacCer species across both serum and urine, including LacCer(d18:1/16:0), (d18:1/22:0) and (d18:1/24:0) (Supplementary Table S5). Furthermore, urinary LacCer(d18:1/16:0), (d18:1/24:0) and (d18:1/24:1) concentrations were approximately twice as high in males as in females, indicating sex-specific differences (Ref. 26). Tissue-level LacCer variations are contrasting from these systemic patterns observed in biofluids. Chatterjee et al. (Ref. 78) examined tissue from polycystic kidney disease (PKD) and reported an increase in LacCer(d18:1/16:0), (d18:1/18:0) and (d18:1/18:1), alongside decreased LacCer(d18:1/24:0) and (d18:1/24:1), and complete absence (d18:1/19:0) and (d18:1/20:0) (Supplementary Table S5), when compared to control samples. These findings indicate either a disease – or matrix-specific reorganisation of LacCer acyl-chain composition within the kidney itself (Ref. 78).

### Cancer and LacCer dysregulation across tissues

Cancer-associated LacCer dysregulation is likely driven by perturbed GSL metabolism and enzymatic remodelling of upstream gangliosides. Experimental studies in colon and renal carcinoma models indicate that increased sialidase NEU3 activity promotes the hydrolysis of gangliosides, including GM_3_ and GD3, leading to LacCer enrichment (Refs 113, 114). Additionally, this shift in NEU3 balance has been associated with reduced apoptotic signalling and enhanced motility-related pathways (Ref. 114). Collectively supporting a functional role for NEU3-linked LacCer remodelling in tumour progression.

Emerging evidence indicates that LacCer profiles are altered across diverse cancer types, reflecting tumour type, stage and progression. In primary colorectal cancer (CC), Procházková et al. (Ref. 65) demonstrated increased LacCer(d18:1/16:0), (d18:1/18:0), (d18:1/22:0), (d18:1/24:0) and (d18:1/24:1) in colon tumour tissue compared with controls (Supplementary Table S6). Complementing these findings, Telleria et al. (Ref. 66) reported that faecal LacCer(d18:1/16:0) discriminates CC from advanced adenoma (Supplementary Table S6), which is suggestive of both local and systemic changes in LacCer metabolism. In hepatic malignancies, cholangiocarcinoma (CCA) tissue shows elevated LacCer(d18:1/16:0) and LacCer (d18:1/h16:0 and d18:1/h24:0, where h denotes a hydroxylated fatty acyl chain), while plasma LacCer(40:3), likely representing (d18:1/22:2), is increased in hepatocellular carcinoma (HCC) (Supplementary Table S6) (Refs 68, 69). In the central nervous system, serum LacCer(d18:1/12:0) is elevated in anaplastic astrocytoma (Supplementary Table S6), whereas in prostate cancer, urinary exosomal LacCer(d18:1/16:0) is increased (Supplementary Table S6) (Refs 67, 70). In haematological malignancies, total LacCer is reduced in bone marrow and plasma from multiple myeloma (MM) compared with asymptomatic monoclonal gammopathy of undetermined significance (MGUS) (Supplementary Table S6) (Ref. 71). In melanoma metastases, total LacCer is also decreased; however, higher circulating LacCer(d18:1/16:0) was inversely associated with mortality (Supplementary Table S6), indicating species-specific associations with disease progression and outcome (Ref. 31).

## Integrated LacCer species patterns across disease contexts

Integration of species-level data throughout this review (Tables 1–3; Supplementary S5–S6) reveals that the broader LacCer profile is characterised by significant heterogeneity or is insufficiently reported across the literature. However, LacCer(d18:1/16:0), (d18:1/24:0) and (d18:1/24:1) demonstrate cross-system trends linking disparate pathologies through convergent inflammatory and metabolic pathways. LacCer(d18:1/16:0) represents the most frequently reported species, and the direction of change appears to be dependent on biological matrix, tissue compartment and disease stage. It is frequently reduced in the circulation in patients with chronic metabolic and renal disorders, likely because it is the immediate precursor of GM_3_(d18:1/16:0), an inhibitor of TLR4 activation. Consequently, depleted LacCer(d18:1/16:0) may drive persistent low-grade inflammation.

In contrast, very-long-chain LacCer species display marked compartmentalisation across disease contexts. LacCer(d18:1/24:0) and (d18:1/24:1) are reduced in circulation in patients with metabolic syndrome, SLE-associated atherosclerosis and CKD. However, they are enriched within localised diseased matrices, such as liver biopsies from NASH patients, atherosclerotic plaques and the urine of individuals with lupus nephritis (Table 2, S Supplementary Tables S3 and S5). This accumulation in local microenvironments suggests these species may contribute to local pathological processes. As established in the immune context (Section ‘LacCers across immune and inflammatory system’), the longer acyl chains of LacCer(d18:1/24:0) and (d18:1/24:1) are structural requirements for stabilising signalling rafts that activate NADPH oxidase. Their localised accumulation in diseased tissues, rather than in the circulation, likely facilitates the persistent production of ROS and subsequent tissue remodelling.

## Limitations

A limitation of this review was the inability to draw definitive conclusions for individual LacCer species due to differing analytical approaches utilised across the literature, including LC–MS/MS methodologies, extraction protocols, species coverage, quantification and reporting methods. Additionally, the use of diverse sample matrices (plasma, serum, urine, tissue and cells) introduces further heterogeneity. Such variability complicates cross-study comparisons, as differences in matrix composition and analytical conditions can influence detection sensitivity and relative abundance measurements. Consequently, whilst we have observed recurring patterns, we have avoided direct species-specific comparisons.

## Conclusion and future directions

This review demonstrates that LacCer is a central intermediate in many common inflammatory and immune-mediated pathways, serving as a link between multiple biological systems. Most studies examined LacCer in the context of specific diseases using untargeted lipidomics or GSL profiling. These approaches have been valuable for discovery, especially since lipidomics has traditionally been a hypothesis-driven field. However, the evidence here suggests a shift of integrating lipidomics into a systems biology framework will enable moving beyond biomarker identification towards clarifying mechanisms. Therefore, future research should adopt integrated multi-omics strategies to link LacCer biology with upstream precursors, downstream products (e.g., GSLs) and associated signalling pathways. Combining lipidomics with transcriptomics and proteomics will enable mechanistic insight beyond association studies and accelerate the identification of therapeutic targets for metabolic, immune and inflammatory disorders.

## Supporting information

10.1017/erm.2026.10054.sm001 Hicks and Whiley supplementary material Hicks and Whiley supplementary material

## Supplementary material

To view supplementary material for this article, please visit http://doi.org/10.1017/erm.2026.10054.

## Long descriptions

### Figure 1. Long description

At the top left is ceramide with its chemical structure. A blue downward arrow labeled U G C G points from ceramide to glucosylceramide at the bottom left, indicating biosynthesis. A red upward arrow labeled G B A points from glucosylceramide back to ceramide, indicating hydrolysis. From glucosylceramide, a blue rightward arrow labeled beta 4 G A L T 5 slash 6 points to lactosylceramide on the right, showing biosynthesis. A red leftward arrow labeled G L B 1 points from lactosylceramide to glucosylceramide, indicating hydrolysis. Each molecule is shown with its chemical structure. At the top right, a legend box contains blue for biosynthesis and red for hydrolysis.

Navigate back to Figure 1..

### Figure 2. Long description

At the center is bold black text Lactosylceramide. Four arrows extend outward: blue arrow (top left) labeled S T 3 G A L 5 points to GM3 a-series, with functions cellular signalling and growth factor receptors, located in neuronal and immune cells, linked to insulin resistance, inflammation, and neurodegenerative pathogenesis. Brown arrow (top right) labeled B4 G A L N T 1 points to GA2 o-series, a precursor for cell-cell recognition, neural development, and synaptic transmission, located in the central nervous system, associated with GM2 accumulation. Red arrow (bottom left) labeled B3 G N T 5 points to LC3 lacto/neolacto-series, involved in cell adhesion, embryonic development, and immune modulation, found in epithelial and immune cells, linked to autoimmune diseases and cancer progression. Green arrow (bottom right) labeled A4 G A L T points to Gb3 globoside, with functions in lipid raft formation and pathogen-host interactions, located in endothelial cells, epithelial tissue, and kidney, associated with Fabry disease and Shiga toxin-producing E. coli. Each branch is color-coded and contains bullet points for function, location, and disease.

Navigate back to Figure 2..

### Table 1. Long description

Beginning at the top row, the table lists eight immune-mediated conditions in the leftmost column: Bacterial vs viral, Pneumonia vs U R I, Acute vs recovered pneumonia, COVID-19 severity, U C, Paediatric U C vs C D (two references), and Paediatric U C/C D. The next column specifies sample matrices, including plasma, serum, urine, and colon tissue. Moving right, each subsequent column represents a distinct LacCer species: d18:1/12:0, d18:1/14:0, d18:1/16:0, d18:1/18:0, d18:1/18:1, d18:1/22:0, d18:1/24:0, and d18:1/24:1. For each condition, the table indicates whether a LacCer species is increased (upward arrow), decreased (downward arrow), not reported (n.r), or not significant (n.s). Increased LacCer (d18:1/16:0) is consistently observed across all conditions except U C (d18:1/18:0, d18:1/24:0 also increased), COVID-19 severity (d18:1/22:0, d18:1/24:0 also increased), and Paediatric U C vs C D (d18:1/14:0 increased in one reference). Most other species are not reported or not significant. The table footnote defines all abbreviations and symbols used.

Navigate back to Table 1..

### Table 3. Long description

Beginning at the top row, the LacCer species d18:1/14:0 shows increased levels in I P D, D L B, A D, P S P, and M S A, with not reported for G B A 1-P D plasma, G B A 1-P D C S F, R R M S, and P M S. The next row, d18:1/16:0, is increased in I P D, D L B, A D, P S P, and M S A, not significant in G B A 1-P D plasma, G B A 1-P D C S F, and R R M S, and increased in P M S. For d18:1/16:1, increased in I P D, D L B, P S P, and M S A, not reported in G B A 1-P D plasma and C S F, not significant in A D and R R M S, and increased in P M S. The d18:1/18:0 species is not significant in I P D and P S P, increased in D L B, A D, M S A, and G B A 1-P D plasma, decreased in G B A 1-P D C S F, not reported in R R M S and P M S. For d18:1/18:1, not significant in I P D, A D, P S P, M S A, G B A 1-P D plasma and C S F, D L B is increased, R R M S and P M S are not significant. The d18:1/20:0 row is not significant in I P D, D L B, A D, P S P, M S A, R R M S, and P M S, decreased in G B A 1-P D plasma and C S F. For d18:1/20:1, not significant in I P D, P S P, M S A, G B A 1-P D plasma and C S F, increased in D L B and A D, decreased in R R M S and P M S. The d18:1/22:0 species is not significant in I P D, D L B, A D, P S P, M S A, G B A 1-P D C S F, R R M S, increased in G B A 1-P D plasma, P M S. For d18:1/22:1, not significant in I P D, P S P, M S A, G B A 1-P D C S F, R R M S, increased in D L B, A D, G B A 1-P D plasma, P M S. The d18:1/22:2 row is not significant in I P D, P S P, M S A, G B A 1-P D plasma and C S F, increased in D L B and A D, not reported in R R M S and P M S. For d18:1/24:0, not significant in I P D, D L B, A D, P S P, G B A 1-P D plasma and C S F, R R M S, P M S, decreased in M S A. The d18:1/24:1 row is not significant in I P D, A D, P S P, M S A, G B A 1-P D plasma and C S F, R R M S, increased in D L B and P M S. For d18:1/24:2, not significant in I P D, A D, P S P, M S A, G B A 1-P D plasma and C S F, R R M S, P M S, increased in D L B. The table footnotes clarify that upward arrows indicate increased versus controls, downward arrows indicate decreased versus controls, n.r means not reported, n.s means no significance reported, and all disorder abbreviations are defined.

Navigate back to Table 3..

### Table 2. Long description

The table columns are Condition, Matrix, and LacCer species (d18:1/12:0, 14:0, 16:0, 18:0, 18:1, 20:0, 22:0, 24:0, 24:1, and 26:0).

Key findings include:

* Overweight (Serum): LacCer (d18:1/12:0) is increased.

* MetS (Serum): LacCer (d18:1/14:0), (d18:1/16:0), and (d18:1/24:1) are decreased.

* IFG (Serum): LacCer (d18:1/12:0) is increased.

* IFG (Plasma): LacCer (d18:1/16:0) is decreased.

* I R plus T 2 D (Plasma/Serum): LacCer (d18:1/14:0), (d18:1/16:0), and (d18:1/24:1) are decreased.

* T 2 D risk (Plasma): Multiple species including (d18:1/14:0), (d18:1/16:0), (d18:1/20:0), (d18:1/22:0), and (d18:1/24:1) are decreased across different studies.

* Obesity plus T 2 D (Adipo-somes): LacCer (d18:1/16:0), (d18:1/18:0), (d18:1/20:0), and (d18:1/24:1) are increased.

* T 2 D (Plasma): LacCer (d18:1/18:0), (d18:1/20:0), (d18:1/24:0), and (d18:1/26:0) are increased.

* T 2 D plus M A (Plasma): LacCer (d18:1/18:0), (d18:1/20:0), (d18:1/24:0), and (d18:1/26:0) are decreased, while (d18:1/22:0) and (d18:1/24:1) are increased.

* N A F L D and N A S H: Show mixed results with increases in (d18:1/14:0) or (d18:1/24:1) and decreases in (d18:1/16:0).

* P C O S (Serum): LacCer (d18:1/16:0) is decreased.

Navigate back to Table 2..

## References

[1] Chatterjee S, Balram A and Li W (2021) Convergence: Lactosylceramide-centric Signaling pathways induce inflammation, oxidative stress, and other phenotypic outcomes. International Journal of Molecular Sciences 22, 1816. 33673027 10.3390/ijms22041816 PMC7917694

[2] Chatterjee S and Pandey A (2008) The yin and Yang of lactosylceramide metabolism: Implications in cell function. Biochimica et Biophysica Acta (BBA) - General Subjects 1780, 370–382. 18077097 10.1016/j.bbagen.2007.08.010

[3] Iwabuchi K, Prinetti A, Sonnino S, Mauri L, Kobayashi T, Ishii K, Kaga N, Murayama K, Kurihara H, Nakayama H, Yoshizaki F, Takamori K, Ogawa H and Nagaoka I (2010) Significance of glycosphingolipid fatty acid chain length on membrane microdomain-mediated signal transduction. FEBS Letters 584, 1642–1652. 19852959 10.1016/j.febslet.2009.10.043

[4] Johnson EL, Heaver SL, Waters JL, Kim BI, Bretin A, Goodman AL, Gewirtz AT, Worgall TS and Ley RE (2020) Sphingolipids produced by gut bacteria enter host metabolic pathways impacting ceramide levels. Nature Communications 11, 2471. 10.1038/s41467-020-16274-w PMC7235224 32424203

[5] Furukawa K, Clausen H and Sato T (2014) UDP-gal: BetaGlcNAc Beta 1,4-galactosyltransferase, polypeptide 2-6; Xylosylprotein Beta 1,4-galactosyltransferase, polypeptide 7 (galactosyltransferase I) (B4GALT2–7). In Taniguchi N, Honke K, Fukuda M, et al. (eds), Handbook of Glycosyltransferases and Related Genes. Tokyo: Springer Japan, pp. 63–72.

[6] Paciotti S, Albi E, Parnetti L and Beccari T (2020) Lysosomal ceramide metabolism disorders: Implications in Parkinson’s disease. Journal of Clinical Medicine 9, 594. 32098196 10.3390/jcm9020594 PMC7073989

[7] Lo N (1998) The expanding beta 4-galactosyltransferase gene family: Messages from the databanks. Glycobiology 8, 517–526. 9597550 10.1093/glycob/8.5.517

[8] Hammad SM, Pierce JS, Soodavar F, Smith KJ, Al Gadban MM, Rembiesa B, Klein RL, Hannun YA, Bielawski J and Bielawska A (2010) Blood sphingolipidomics in healthy humans: Impact of sample collection methodology. Journal of Lipid Research 51, 3074–3087. 20660127 10.1194/jlr.D008532 PMC2936747

[9] Scherer M, Böttcher A, Schmitz G and Liebisch G (2011) Sphingolipid profiling of human plasma and FPLC-separated lipoprotein fractions by hydrophilic interaction chromatography tandem mass spectrometry. Biochimica et Biophysica Acta (BBA) - Molecular and Cell Biology of Lipids 1811, 68–75. 21081176 10.1016/j.bbalip.2010.11.003

[10] Kumpula LS, Kumpula JM, Taskinen M-R, Jauhiainen M, Kaski K and Ala-Korpela M (2008) Reconsideration of hydrophobic lipid distributions in lipoprotein particles. Chemistry and Physics of Lipids 155, 57–62. 18611396 10.1016/j.chemphyslip.2008.06.003

[11] Hülsmeier AJ (2025) Glycosphingolipids in neurodegeneration – Molecular mechanisms, cellular roles, and therapeutic perspectives. Neurobiology of Disease 207, 106851. 39978484 10.1016/j.nbd.2025.106851

[12] Chiricozzi E, Lunghi G, Valsecchi M, Carsana EV, Bassi R, Di Biase E, Dobi D, Ciampa MG, Mauri L, Aureli M, Inamori K, Inokuchi J, Sonnino S and Fazzari M (2025) Metabolic and structural consequences of GM3 synthase deficiency: Insights from an HEK293-T knockout model. Biomedicine 13, 843. 10.3390/biomedicines13040843 PMC12024672 40299395

[13] Inokuchi J, Nagafuku M, Ohno I and Suzuki A (2013) Heterogeneity of gangliosides among T cell subsets. Cellular and Molecular Life Sciences 70, 3067–3075. 23233133 10.1007/s00018-012-1208-x PMC11114073

[14] Kanoh H, Nitta T, Go S, Inamori K, Veillon L, Nihei W, Fujii M, Kabayama K, Shimoyama A, Fukase K, Ohto U, Shimizu T, Watanabe T, Shindo H, Aoki S, Sato K, Nagasaki M, Yatomi Y, Komura N, … Inokuchi J (2020) Homeostatic and pathogenic roles of GM 3 ganglioside molecular species in TLR 4 signaling in obesity. The EMBO Journal 39, e101732. 32378734 10.15252/embj.2019101732 PMC7298289

[15] Rajesh M, Kolmakova A and Chatterjee S (2005) Novel role of Lactosylceramide in vascular endothelial growth factor–mediated angiogenesis in human endothelial cells. Circulation Research 97, 796–804. 16151023 10.1161/01.RES.0000185327.45463.A8

[16] Chatterjee S, Clarke KS and Kwiterovich PO (1986) Uptake and metabolism of lactosylceramide on low density lipoproteins in cultured proximal tubular cells from normal and familial hypercholesterolemic homozygotes. The Journal of Biological Chemistry 261, 13480–13486. 3759974

[17] Hůlková H, Ledvinová J, Asfaw B, Koubek K, Kopřiva K and Elleder M (2005) Lactosylceramide in lysosomal storage disorders. A comparative immunohistochemical and biochemical study. Virchows Archiv 447, 31–44. 15918012 10.1007/s00428-005-1246-y

[18] Stevens CR, Oberholzer VG, Walker-Smith JA and Phillips AD (1988) Lactosylceramide in inflammatory bowel disease: A biochemical study. Gut 29, 580–587. 3396945 10.1136/gut.29.5.580 PMC1433632

[19] Veldman RJ, Klappe K, Hinrichs J, Hummel I, Schaaf G, Sietsma H and Kok JW (2002) Altered sphingolipid metabolism in multidrug-resistant ovarian cancer cells is due to uncoupling of glycolipid biosynthesis in the Golgi apparatus. The FASEB Journal 16, 1111–1113. 12039850 10.1096/fj.01-0863fje

[20] Bodas M, Min T and Vij N (2015) Lactosylceramide-accumulation in lipid-rafts mediate aberrant-autophagy, inflammation and apoptosis in cigarette smoke induced emphysema. Apoptosis: An International Journal on Programmed Cell Death 20, 725–739. 25638276 10.1007/s10495-015-1098-0

[21] Mukhin DN, Chao F-F and Kruth HS (1995) Glycosphingolipid accumulation in the Aortic Wall is another feature of human atherosclerosis. Arteriosclerosis, Thrombosis, and Vascular Biology 15, 1607–1615. 7583534 10.1161/01.atv.15.10.1607

[22] Hammad SM, Harden OC, Wilson DA, Twal WO, Nietert PJ and Oates JC (2021) Plasma sphingolipid profile associated with subclinical atherosclerosis and clinical disease markers of systemic lupus erythematosus: Potential predictive value. Frontiers in Immunology 12, 694318. 34367153 10.3389/fimmu.2021.694318 PMC8335560

[23] Apostolopoulou M, Gordillo R, Koliaki C, Gancheva S, Jelenik T, De Filippo E, Herder C, Markgraf D, Jankowiak F, Esposito I, Schlensak M, Scherer PE and Roden M (2018) Specific hepatic sphingolipids relate to insulin resistance, oxidative stress, and inflammation in nonalcoholic steatohepatitis. Diabetes Care 41, 1235–1243. 29602794 10.2337/dc17-1318

[24] Shui G, Lam SM, Stebbins J, Kusunoki J, Duan X, Li B, Cheong WF, Soon D, Kelly RP and Wenk MR (2013) Polar lipid derangements in type 2 diabetes mellitus: Potential pathological relevance of fatty acyl heterogeneity in sphingolipids. Metabolomics 9, 786–799.

[25] Wilmott LA, Grambergs RC, Allegood JC, Lyons TJ and Mandal N (2019) Analysis of sphingolipid composition in human vitreous from control and diabetic individuals. Journal of Diabetes and its Complications 33, 195–201. 30630661 10.1016/j.jdiacomp.2018.12.005 PMC6368445

[26] Wolf B, Blaschke CRK, Mungaray S, Weselman BT, Stefanenko M, Fedoriuk M, Bai H, Rodgers J, Palygin O, Drake RR and Nowling TK (2023) Metabolic markers and Association of Biological sex in lupus nephritis. International Journal of Molecular Sciences 24, 16490. 38003679 10.3390/ijms242216490 PMC10671813

[27] Yau A, Fear MW, Gray N, Ryan M, Holmes E, Nicholson JK, Whiley L and Wood FM (2022) Enhancing the accuracy of surgical wound excision following burns trauma via application of rapid evaporative IonisationMass spectrometry (REIMS). Burns 48, 1574–1583. 36116996 10.1016/j.burns.2022.08.021

[28] Noel A, Ingrand S and Barrier L (2017) Ganglioside and related-sphingolipid profiles are altered in a cellular model of Alzheimer’s disease. Biochimie 137, 158–164. 28365364 10.1016/j.biochi.2017.03.019

[29] Oizumi H, Sugimura Y, Totsune T, Kawasaki I, Ohshiro S, Baba T, Kimpara T, Sakuma H, Hasegawa T, Kawahata I, Fukunaga K and Takeda A (2022) Plasma sphingolipid abnormalities in neurodegenerative diseases. PLoS One 17, e0279315. 36525454 10.1371/journal.pone.0279315 PMC9757566

[30] Liu D, Aziz NA, Landstra EN and Breteler MMB (2023) The lipidomic correlates of epigenetic aging across the adult lifespan: A population-based study. Aging Cell 22. 10.1111/acel.13934 PMC10497837 37496173

[31] Szász I, Koroknai V, Várvölgyi T, Pál L, Szűcs S, Pikó P, Emri G, Janka E, Szabó IL, Ádány R and Balázs M (2024) Identification of plasma lipid alterations associated with melanoma metastasis. International Journal of Molecular Sciences 25, 4251. 38673837 10.3390/ijms25084251 PMC11050015

[32] Muilwijk M, Goorden SMI, Celis-Morales C, Hof MH, Ghauharali-van Der Vlugt K, Beers-Stet FS, Gill JMR, Vaz FM and Van Valkengoed IGM (2020) Contributions of amino acid, acylcarnitine and sphingolipid profiles to type 2 diabetes risk among south-Asian Surinamese and Dutch adults. BMJ Open Diabetes Research & Care 8, e001003. 10.1136/bmjdrc-2019-001003 PMC7228466 32376636

[33] Han X (2016) Lipidomics for studying metabolism. Nature Reviews Endocrinology 12, 668–679. 10.1038/nrendo.2016.98 27469345

[34] Hammad SM, Hardin JR, Wilson DA, Twal WO, Nietert PJ and Oates JC (2019) Race disparity in blood sphingolipidomics associated with lupus cardiovascular comorbidity. PLoS One 14, e0224496. 31747417 10.1371/journal.pone.0224496 PMC6867606

[35] Fiorelli S, Anesi A, Porro B, Cosentino N, Werba JP, Di Minno A, Manega CM, Barbieri S, Colombo GI, Marenzi G, Cavalca V, Tremoli E and Eligini S (2021) Lipidomics analysis of monocytes from patients with acute myocardial infarction reveals lactosylceramide as a new player in monocyte migration. The FASEB Journal 35. 10.1096/fj.202001872RRR 33856696

[36] Edsfeldt A, Dunér P, Ståhlman M, Mollet IG, Asciutto G, Grufman H, Nitulescu M, Persson AF, Fisher RM, Melander O, Orho-Melander M, Borén J, Nilsson J and Gonçalves I (2016) Sphingolipids contribute to human atherosclerotic plaque inflammation. Arteriosclerosis, Thrombosis, and Vascular Biology 36, 1132–1140. 27055903 10.1161/ATVBAHA.116.305675

[37] Berkowitz L, Salazar C, Ryff CD, Coe CL and Rigotti A (2022) Serum sphingolipid profiling as a novel biomarker for metabolic syndrome characterization. Frontiers in Cardiovascular Medicine 9, 1092331. 36578837 10.3389/fcvm.2022.1092331 PMC9791223

[38] Neb H, Roth V, Roos J, Bauer T, Urbschat A, Heinicke U, Angioni C, Steinhilber D, Piesche M, Ferreirós N, Gurke R, Geisslinger G, Utech E, Zacharowski K, Meybohm P, Paulus P, Schmitt E and Maier TJ (2025) Analysis of fatty acid-derived lipids in critically ill patients after cardiac surgery yields novel pathophysiologically relevant mediators with possible relevance for systemic inflammatory reactions. Frontiers in Immunology 15, 1148806. 39959584 10.3389/fimmu.2024.1148806 PMC11826806

[39] Chatterjee SB, Dey S, Shi WY, Thomas K and Hutchins GM (1997) Accumulation of glycosphingolipids in human atherosclerotic plaque and unaffected aorta tissues. Glycobiology 7, 57–65. 9061365 10.1093/glycob/7.1.57

[40] Zdanowicz K, Bobrus-Chcociej A, Pogodzinska K, Blachnio-Zabielska A, Zelazowska-Rutkowska B, Lebensztejn DM and Daniluk U (2022) Analysis of sphingolipids in pediatric patients with cholelithiasis – A preliminary study. Journal of Clinical Medicine 11, 5613. 36233480 10.3390/jcm11195613 PMC9570855

[41] Kim M, Jung S, Lee S-H and Lee JH (2015) Association between arterial stiffness and serum L-Octanoylcarnitine and Lactosylceramide in overweight middle-aged subjects: 3-year follow-up study. PLoS One 10, e0119519. 25781947 10.1371/journal.pone.0119519 PMC4363527

[42] Rigamonti AE, Dei Cas M, Caroli D, De Col A, Cella SG, Paroni R and Sartorio A (2023) Identification of a specific plasma sphingolipid profile in a Group of Normal-Weight and Obese Subjects: A novel approach for a “biochemical diagnosis of metabolic syndrome?. International Journal of Molecular Sciences 24, 7451. 37108620 10.3390/ijms24087451 PMC10138812

[43] Jung S, Kim M, Lee YJ, Lee S and Lee JH (2015) Associations between metabolomic-identified changes of biomarkers and arterial stiffness in subjects progressing to impaired fasting glucose. Clinical Endocrinology 83, 196–204. 25990250 10.1111/cen.12821

[44] Jensen PN, Fretts AM, Yu C, Hoofnagle AN, Umans JG, Howard BV, Sitlani CM, Siscovick DS, King IB, Sotoodehnia N, McKnight B and Lemaitre RN (2019) Circulating sphingolipids, fasting glucose, and impaired fasting glucose: The strong heart family study. eBioMedicine 41, 44–49. 30594552 10.1016/j.ebiom.2018.12.046 PMC6444022

[45] Berkowitz L, Razquin C, Salazar C, Biancardi F, Estruch R, Ros E, Fitó M, Corella D, Coe CL, Ryff CD, Ruiz-Canela M, Salas-Salvado J, Wang D, Hu FB, Deik A, Martínez-Gonzalez MA and Rigotti A (2024) Sphingolipid profiling as a biomarker of type 2 diabetes risk: Evidence from the MIDUS and PREDIMED studies. Cardiovascular Diabetology 23, 446. 39695759 10.1186/s12933-024-02505-7 PMC11657495

[46] Perreault L, Starling AP, Glueck D, Brozinick JT, Sanders P, Siddall P, Kuo MS, Dabelea D and Bergman BC (2016) Biomarkers of ectopic fat deposition: The next frontier in serum lipidomics. The Journal of Clinical Endocrinology & Metabolism 101, 176–182. 26574956 10.1210/jc.2015-3213 PMC4701843

[47] Lai M, Al Rijjal D, Röst HL, Dai FF, Gunderson EP and Wheeler MB (2020) Underlying dyslipidemia postpartum in women with a recent GDM pregnancy who develop type 2 diabetes. eLife 9, e59153. 32748787 10.7554/eLife.59153 PMC7417169

[48] Fretts AM, Jensen PN, Hoofnagle A, McKnight B, Howard BV, Umans J, Yu C, Sitlani C, Siscovick DS, King IB, Sotoodehnia N and Lemaitre RN (2020) Plasma ceramide species are associated with diabetes risk in participants of the strong heart study. The Journal of Nutrition 150, 1214–1222. 31665380 10.1093/jn/nxz259 PMC7198314

[49] Mirza I, Haloul M, Hassan C, Masrur M, Mostafa A, Bianco FM, Ali MM, Minshall RD and Mahmoud AM (2023) Adiposomes from obese-diabetic individuals promote endothelial dysfunction and loss of surface caveolae. Cells 12, 2453. 37887297 10.3390/cells12202453 PMC10605845

[50] Hammad SM, Hunt KJ, Baker NL, Klein RL and Lopes-Virella MF (2022) Diabetes and kidney dysfunction markedly alter the content of sphingolipids carried by circulating lipoproteins. Journal of Clinical Lipidology 16, 173–183. 35148982 10.1016/j.jacl.2021.12.004 PMC12882810

[51] Lopes-Virella MF, Hammad SM, Baker NL, Klein RL and Hunt KJ (2024) Circulating lipoprotein sphingolipids in chronic kidney disease with and without diabetes. Biomedicine 12, 190. 10.3390/biomedicines12010190 PMC10813484 38255295

[52] Gadgil MD, Sarkar M, Sands C, Lewis MR, Herrington DM and Kanaya AM (2022) Associations of NAFLD with circulating ceramides and impaired glycemia. Diabetes Research and Clinical Practice 186, 109829. 35292328 10.1016/j.diabres.2022.109829 PMC9082931

[53] Faquih TO, Van Klinken JB, Li‐Gao R, Noordam R, Van Heemst D, Boone S, Sheridan PA, Michelotti G, Lamb H, De Mutsert R, Rosendaal FR, Van Hylckama Vlieg A, Van Dijk KW and Mook‐Kanamori DO (2023) Hepatic triglyceride content is intricately associated with numerous metabolites and biochemical pathways. Liver International 43, 1458–1472. 37017544 10.1111/liv.15575

[54] Getz KR, Jeon MS, Luo C, Luo J and Toriola AT (2023) Lipidome of mammographic breast density in premenopausal women. Breast Cancer Research 25. 10.1186/s13058-023-01725-1 PMC10561435 37814330

[55] Getz KR, Jeon MS, Liu L, Liu L, Zhang H, Luo C, Luo J and Toriola AT (2025) Metabolites and lipid species mediate the associations of adiposity in childhood and early adulthood with mammographic breast density in premenopausal women. Breast Cancer Research 27. 10.1186/s13058-025-01970-6 PMC11796188 39905412

[56] Li J, Xie L-M, Song J-L, Yau L-F, Mi J-N, Zhang C-R, Wu W-T, Lai M-H, Jiang Z-H, Wang J-R and Ma H-X (2019) Alterations of sphingolipid metabolism in different types of polycystic ovary syndrome. Scientific Reports 9, 3204. 30824725 10.1038/s41598-019-38944-6 PMC6397209

[57] Kierath E, Ryan M, Holmes E, Nicholson JK, Fear MW, Wood FM, Whiley L and Gray N (2023) Plasma lipidomics reveal systemic changes persistent throughout early life following a childhood burn injury. Burns & Trauma 11. 10.1093/burnst/tkad044 PMC10703495 38074192

[58] Wang X, Nijman R, Camuzeaux S, Sands C, Jackson H, Kaforou M, Emonts M, Herberg JA, Maconochie I, Carrol ED, Paulus SC, Zenz W, Van Der Flier M, De Groot R, Martinon-Torres F, Schlapbach LJ, Pollard AJ, Fink C, Kuijpers TT, … Žukovskaja V (2019) Plasma lipid profiles discriminate bacterial from viral infection in febrile children. Scientific Reports 9. 10.1038/s41598-019-53721-1 PMC6881435 31776453

[59] Wei T, Xu W, Tu B, Zhang W, Yang X, Zhou Y, Zhang S, Yang J, Xie M, Du J, Chen W and Lu Q (2024) Plasma metabonomics of human adenovirus-infected patients with pneumonia and upper respiratory tract infection. Current Medical Science 44, 121–133. 38393525 10.1007/s11596-024-2835-9

[60] Uranbileg B, Isago H, Nakayama H, Jubishi D, Okamoto K, Sakai E, Kubota M, Tsutsumi T, Moriya K and Kurano M (2024) Comprehensive metabolic modulations of sphingolipids are promising severity indicators in COVID-19. The FASEB Journal 38. 10.1096/fj.202401099R 39012295

[61] Daniluk U, Daniluk J, Kucharski R, Kowalczyk T, Pietrowska K, Samczuk P, Filimoniuk A, Kretowski A, Lebensztejn D and Ciborowski M (2019) Untargeted metabolomics and inflammatory markers profiling in children with Crohn’s disease and ulcerative colitis – A preliminary study. Inflammatory Bowel Diseases 25, 1120–1128. 30772902 10.1093/ibd/izy402

[62] Filimoniuk A, Blachnio-Zabielska A, Imierska M, Lebensztejn DM and Daniluk U (2020) Sphingolipid analysis indicate lactosylceramide as a potential biomarker of inflammatory bowel disease in children. Biomolecules 10, 1083. 32708181 10.3390/biom10071083 PMC7408557

[63] Salihovic S, Nyström N, Mathisen CB-W, Kruse R, Olbjørn C, Andersen S, Noble AJ, Dorn-Rasmussen M, Bazov I, Perminow G, Opheim R, Detlie TE, Huppertz-Hauss G, Hedin CRH, Carlson M, Öhman L, Magnusson MK, Keita ÅV, Söderholm JD, … Halfvarson J (2024) Identification and validation of a blood- based diagnostic lipidomic signature of pediatric inflammatory bowel disease. Nature Communications 15. 10.1038/s41467-024-48763-7 PMC11148148 38830848

[64] Bazarganipour S, Hausmann J, Oertel S, El-Hindi K, Brachtendorf S, Blumenstein I, Kubesch A, Sprinzl K, Birod K, Hahnefeld L, Trautmann S, Thomas D, Herrmann E, Geisslinger G, Schiffmann S and Grösch S (2019) The lipid status in patients with ulcerative colitis: Sphingolipids are disease-dependent regulated. Journal of Clinical Medicine 8, 971. 31277430 10.3390/jcm8070971 PMC6678307

[65] Procházková J, Slavík J, Bouchal J, Levková M, Hušková Z, Ehrmann J, Ovesná P, Kolář Z, Skalický P, Straková N, Zapletal O, Kozubík A, Hofmanová J, Vondráček J and Machala M (2020) Specific alterations of sphingolipid metabolism identified in EpCAM-positive cells isolated from human colon tumors. Biochimica et Biophysica Acta (BBA) - Molecular and Cell Biology of Lipids 1865, 158742. 32447053 10.1016/j.bbalip.2020.158742

[66] Telleria O, Alboniga OE, Clos-Garcia M, Nafría-Jimenez B, Cubiella J, Bujanda L and Falcón-Pérez JM (2022) A comprehensive metabolomics analysis of fecal samples from advanced adenoma and colorectal cancer patients. Metabolites 12, 550. 35736483 10.3390/metabo12060550 PMC9229737

[67] Du C, Huang Z, Wei B and Li M (2022) Comprehensive metabolomics study on the pathogenesis of anaplastic astrocytoma via UPLC-Q/TOF-MS. Medicine 101, e29594. 35945752 10.1097/MD.0000000000029594 PMC9351860

[68] Silsirivanit A, Phoomak C, Teeravirote K, Wattanavises S, Seubwai W, Saengboonmee C, Zhan Z, Inokuchi J, Suzuki A and Wongkham S (2019) Overexpression of HexCer and LacCer containing 2-hydroxylated fatty acids in cholangiocarcinoma and the association of the increase of LacCer (d18:1-h23:0) with shorter survival of the patients. Glycoconjugate Journal 36, 103–111. 30888588 10.1007/s10719-019-09864-4

[69] Yang L, Bai Y, Han X, Shi Y and Liu H (2017) Plasma lipidomic analysis to identify novel biomarkers for hepatocellular carcinoma. Journal of Analysis and Testing 1, 223–232.

[70] Skotland T, Ekroos K, Kauhanen D, Simolin H, Seierstad T, Berge V, Sandvig K and Llorente A (2017) Molecular lipid species in urinary exosomes as potential prostate cancer biomarkers. European Journal of Cancer 70, 122–132. 27914242 10.1016/j.ejca.2016.10.011

[71] Gonsalves WI, Broniowska K, Jessen E, Petterson X-M, Bush AG, Gransee J, Lacy MQ, Hitosugi T and Kumar SK (2020) Metabolomic and lipidomic profiling of bone marrow plasma differentiates patients with monoclonal gammopathy of undetermined significance from multiple myeloma. Scientific Reports 10, 10250. 32581232 10.1038/s41598-020-67105-3 PMC7314797

[72] Filippatou AG, Moniruzzaman M, Sotirchos ES, Fitzgerald KC, Kalaitzidis G, Lambe J, Vasileiou E, Saidha S, Prince JL, Haughey N, Calabresi PA and Bhargava P (2021) Serum ceramide levels are altered in multiple sclerosis. Multiple Sclerosis Journal 27, 1506–1519. 33307993 10.1177/1352458520971816 PMC8200368

[73] Wang X, Bui H, Vemuri P, Graff-Radford J, Jack CR Jr, Petersen RC and Mielke MM (2021) Lipidomic network of mild cognitive impairment from the Mayo Clinic study of aging. Journal of Alzheimer’s Disease 81, 533–543. 10.3233/JAD-201347 PMC8154710 33814434

[74] Mielke MM, Bandaru VVR, Haughey NJ, Xia J, Fried LP, Yasar S, Albert M, Varma V, Harris G, Schneider EB, Rabins PV, Bandeen-Roche K, Lyketsos CG and Carlson MC (2012) Serum ceramides increase the risk of Alzheimer disease: The women’s health and aging study II. Neurology 79, 633–641. 22815558 10.1212/WNL.0b013e318264e380 PMC3414665

[75] Den Heijer JM, Cullen VC, Pereira DR, Yavuz Y, De Kam ML, Grievink HW, Moerland M, Leymarie N, Khatri K, Sollomoni I, Spitalny L, Dungeon L, Hilt DC, Justman C, Lansbury P and Groeneveld GJ (2023) A biomarker study in patients with *GBA1*-Parkinson’s disease and healthy controls. Movement Disorders 38, 783–795. 36916660 10.1002/mds.29360

[76] the CKiD study group, Mitsnefes M, Scherer PE, Friedman LA, Gordillo R, Furth S and Warady BA (2014) Ceramides and cardiac function in children with chronic kidney disease. Pediatric Nephrology 29, 415–422. 24389650 10.1007/s00467-013-2642-1 PMC4068150

[77] Nowling TK, Mather AR, Thiyagarajan T, Hernández-Corbacho MJ, Powers TW, Jones EE, Snider AJ, Oates JC, Drake RR and Siskind LJ (2015) Renal glycosphingolipid metabolism is dysfunctional in lupus nephritis. Journal of the American Society of Nephrology 26, 1402–1413. 25270066 10.1681/ASN.2014050508 PMC4446878

[78] Chatterjee S, Shi WY, Wilson P and Mazumdar A (1996) Role of lactosylceramide and MAP kinase in the proliferation of proximal tubular cells in human polycystic kidney disease. Journal of Lipid Research 37, 1334–1344. 8808768

[79] Quinn RA, Phelan VV, Whiteson KL, Garg N, Bailey BA, Lim YW, Conrad DJ, Dorrestein PC and Rohwer FL (2016) Microbial, host and xenobiotic diversity in the cystic fibrosis sputum metabolome. The ISME Journal 10, 1483–1498. 26623545 10.1038/ismej.2015.207 PMC5029181

[80] Boenzi S, Catesini G, Sacchetti E, Tagliaferri F, Dionisi-Vici C and Deodato F (2021) Comprehensive-targeted lipidomic analysis in Niemann-Pick C disease. Molecular Genetics and Metabolism 134, 337–343. 34810067 10.1016/j.ymgme.2021.11.005

[81] Arai T, Bhunia AK, Chatterjee S and Bulkley GB (1998) Lactosylceramide stimulates human neutrophils to upregulate mac-1, adhere to endothelium, and generate reactive oxygen metabolites in vitro. Circulation Research 82, 540–547. 9529158 10.1161/01.res.82.5.540

[82] Yu W, Ying J, Wang X, Liu X, Zhao T, Yoon S, Zheng Q, Fang Y, Yang D and Hua F (2021) The involvement of lactosylceramide in central nervous system inflammation related to neurodegenerative disease. Frontiers in Aging Neuroscience 13, 691230. 34349634 10.3389/fnagi.2021.691230 PMC8326838

[83] Couto D, Santinha D, Melo T, Ferreira-Fernandes E, Videira RA, Campos A, Fardilha M, Domingues P and Domingues MRM (2015) Glycosphingolipids and oxidative stress: Evaluation of hydroxyl radical oxidation of galactosyl and lactosylceramides using mass spectrometry. Chemistry and Physics of Lipids 191, 106–114. 26315528 10.1016/j.chemphyslip.2015.08.014

[84] Nakayama H, Nagafuku M, Suzuki A, Iwabuchi K and Inokuchi J (2018) The regulatory roles of glycosphingolipid-enriched lipid rafts in immune systems. FEBS Letters 592, 3921–3942. 30320884 10.1002/1873-3468.13275

[85] Nagafuku M, Okuyama K, Onimaru Y, Suzuki A, Odagiri Y, Yamashita T, Iwasaki K, Fujiwara M, Takayanagi M, Ohno I and Inokuchi J (2012) CD4 and CD8 T cells require different membrane gangliosides for activation. Proceedings of the National Academy of Sciences 109, E336–E342. 10.1073/pnas.1114965109 PMC3277553 22308377

[86] Soehnlein O (2012) Multiple roles for neutrophils in atherosclerosis. Circulation Research 110, 875–888. 22427325 10.1161/CIRCRESAHA.111.257535

[87] Meng L-B, Yu Z-M, Guo P, Wang Q-Q, Qi R-M, Shan M-J, Lv J and Gong T (2018) Neutrophils and neutrophil-lymphocyte ratio: Inflammatory markers associated with intimal-media thickness of atherosclerosis. Thrombosis Research 170, 45–52. 30118867 10.1016/j.thromres.2018.08.002

[88] Iwabuchi K, Prinetti A, Sonnino S, Mauri L, Kobayashi T, Ishii K, Kaga N, Murayama K, Kurihara H, Nakayama H, Yoshizaki F, Takamori K, Ogawa H and Nagaoka I (2008) Involvement of very long fatty acid-containing lactosylceramide in lactosylceramide-mediated superoxide generation and migration in neutrophils. Glycoconjugate Journal 25, 357–374. 18041581 10.1007/s10719-007-9084-6

[89] Chiricozzi E, Ciampa MG, Brasile G, Compostella F, Prinetti A, Nakayama H, Ekyalongo RC, Iwabuchi K, Sonnino S and Mauri L (2015) Direct interaction, instrumental for signaling processes, between LacCer and Lyn in the lipid rafts of neutrophil-like cells. Journal of Lipid Research 56, 129–141. 25418321 10.1194/jlr.M055319 PMC4274061

[90] Powell DW, Brady M, Tandon S, Lightman R, Short NA, Rane MJ, Barati MT and Caster DJ (2023) Neutrophil degranulation in lupus nephritis: SA-PO845. Journal of the American Society of Nephrology 34, 962–963.

[91] Wentworth JM, Naselli G, Ngui K, Smyth GK, Liu R, O’Brien PE, Bruce C, Weir J, Cinel M, Meikle PJ and Harrison LC (2016) GM3 ganglioside and phosphatidylethanolamine-containing lipids are adipose tissue markers of insulin resistance in obese women. International Journal of Obesity 40, 706–713. 26499445 10.1038/ijo.2015.223

[92] Yamashita T (2011) Glycosphingolipid modification: Structural diversity, functional and mechanistic integration of diabetes. Diabetes & Metabolism Journal 35, 309–316. 21977449 10.4093/dmj.2011.35.4.309 PMC3178690

[93] Dam DHM, Wang X-Q, Sheu S, Vijay M, Shipp D, Miller L and Paller AS (2017) Ganglioside GM3 mediates glucose-induced suppression of IGF-1 receptor-Rac1 activation to inhibit keratinocyte motility. The Journal of Investigative Dermatology 137, 440–448. 27729281 10.1016/j.jid.2016.09.028 PMC5499673

[94] Gong N, Wei H, Chowdhury SH and Chatterjee S (2004) Lactosylceramide recruits PKCalpha/epsilon and phospholipase A2 to stimulate PECAM-1 expression in human monocytes and adhesion to endothelial cells. Proceedings of the National Academy of Sciences of the United States of America 101, 6490–6495. 15084746 10.1073/pnas.0308684101 PMC404072

[95] Hamilton JA, Hillard CJ, Spector AA and Watkins PA (2007) Brain uptake and utilization of fatty acids, lipids and lipoproteins: Application to neurological disorders. Journal of Molecular Neuroscience 33, 2–11. 17901539 10.1007/s12031-007-0060-1

[96] Rusciani A, Duca L, Sartelet H, Chatron-Colliet A, Bobichon H, Ploton D, Le Naour R, Blaise S, Martiny L and Debelle L (2010) Elastin peptides Signaling relies on Neuraminidase-1-dependent lactosylceramide generation. PLoS One 5, e14010. 21103358 10.1371/journal.pone.0014010 PMC2982818

[97] Scandolera A, Rabenoelina F, Chaintreuil C, Rusciani A, Maurice P, Blaise S, Romier-Crouzet B, El Btaouri H, Martiny L, Debelle L and Duca L (2015) Uncoupling of elastin complex receptor during in vitro aging is related to modifications in its intrinsic sialidase activity and the subsequent lactosylceramide production. PLoS One 10, e0129994. 26086247 10.1371/journal.pone.0129994 PMC4473072

[98] Grassi S, Giussani P, Mauri L, Prioni S, Sonnino S and Prinetti A (2020) Lipid rafts and neurodegeneration: Structural and functional roles in physiologic aging and neurodegenerative diseases. Journal of Lipid Research 61, 636–654. 31871065 10.1194/jlr.TR119000427 PMC7193971

[99] Zhang S, Gao Z, Feng L and Li M (2024) Prevention and treatment strategies for Alzheimer’s disease: Focusing on microglia and astrocytes in neuroinflammation. Journal of Inflammation Research 17, 7235–7259. 39421566 10.2147/JIR.S483412 PMC11484773

[100] Alzarea SI (2025) Identification of novel neuraminidase 1 modulators as potential therapeutics for Alzheimer’s disease using virtual screening and molecular dynamics simulations. Scientific Reports 15, 39901. 41238601 10.1038/s41598-025-22955-7 PMC12618679

[101] Fedorova TD, Knudsen K, Horsager J, Hansen AK, Okkels N, Gottrup H, Vang K and Borghammer P (2023) Dopaminergic dysfunction is more symmetric in dementia with Lewy bodies compared to Parkinson’s disease. Journal of Parkinson’s Disease 13, 515–523. 10.3233/JPD-230001 PMC10357144 37212074

[102] Caminiti SP, Pilotto A, Premi E, Galli A, Ferrari E, Gipponi S, Cottini E, Paghera B, Perani D and Padovani A (2023) Dopaminergic connectivity reconfiguration in the dementia with Lewy bodies continuum. Parkinsonism & Related Disorders 108, 105288. 36724569 10.1016/j.parkreldis.2023.105288

[103] Greally S, Kumar M, Schlaffner C, Van Der Heijden H, Lawton ES, Biswas D, Berretta S, Steen H and Steen JA (2024) Dementia with lewy bodies patients with high tau levels display unique proteome profiles. Molecular Neurodegeneration 19, 98. 39696638 10.1186/s13024-024-00782-0 PMC11657859

[104] Skrahin A, Horowitz M, Istaiti M, Skrahina V, Lukas J, Yahalom G, Cohen ME, Revel-Vilk S, Goker-Alpan O, Becker-Cohen M, Hassin-Baer S, Svenningsson P, Rolfs A and Zimran A (2024) GBA1-associated Parkinson’s disease is a distinct entity. International Journal of Molecular Sciences 25, 7102. 39000225 10.3390/ijms25137102 PMC11241486

[105] Reddy K and Dieriks BV (2022) Multiple system atrophy: α-synuclein strains at the neuron-oligodendrocyte crossroad. Molecular Neurodegeneration 17, 77. 36435784 10.1186/s13024-022-00579-z PMC9701437

[106] Zhang H, Wei W, Zhao M, Ma L, Jiang X, Pei H, Cao Y and Li H (2021) Interaction between Aβ and tau in the pathogenesis of Alzheimer’s disease. International Journal of Biological Sciences 17, 2181–2192. 34239348 10.7150/ijbs.57078 PMC8241728

[107] Jackson RJ, Melloni A, Fykstra DP, Serrano-Pozo A, Shinobu L and Hyman BT (2024) Astrocyte tau deposition in progressive supranuclear palsy is associated with dysregulation of MAPT transcription. Acta Neuropathologica Communications 12, 132. 39138580 10.1186/s40478-024-01844-6 PMC11323491

[108] Boutitah-Benyaich I, Eixarch H, Villacieros-Álvarez J, Hervera A, Cobo-Calvo Á, Montalban X and Espejo C (2025) Multiple sclerosis: Molecular pathogenesis and therapeutic intervention. Signal Transduction and Targeted Therapy 10, 324. 41034190 10.1038/s41392-025-02415-4 PMC12488951

[109] Florens N, Calzada C, Lyasko E, Juillard L and Soulage C (2016) Modified lipids and lipoproteins in chronic kidney disease: A new class of uremic toxins. Toxins 8, 376. 27999257 10.3390/toxins8120376 PMC5198570

[110] Balram A, Thapa S and Chatterjee S (2022) Glycosphingolipids in diabetes, oxidative stress, and cardiovascular disease: Prevention in experimental animal models. International Journal of Molecular Sciences 23, 15442. 36499769 10.3390/ijms232315442 PMC9735750

[111] Wilson PD, Du J and Norman JT (1993) Autocrine, endocrine and paracrine regulation of growth abnormalities in autosomal dominant polycystic kidney disease. European Journal of Cell Biology 61, 131–138. 8223698

[112] Lopes-Virella MF, Baker NL, Hunt KJ, Hammad SM, Arthur J, Virella G and Klein RL (2019) Glycosylated sphingolipids and progression to kidney dysfunction in type 1 diabetes. Journal of Clinical Lipidology 13, 481–491.e1. 31043336 10.1016/j.jacl.2019.03.005 PMC7218687

[113] Kakugawa Y, Wada T, Yamaguchi K, Yamanami H, Ouchi K, Sato I and Miyagi T (2002) Up-regulation of plasma membrane-associated ganglioside sialidase (Neu3) in human colon cancer and its involvement in apoptosis suppression. Proceedings of the National Academy of Sciences 99, 10718–10723. 10.1073/pnas.152597199 PMC125023 12149448

[114] Ueno S, Saito S, Wada T, Yamaguchi K, Satoh M, Arai Y and Miyagi T (2006) Plasma membrane-associated sialidase is up-regulated in renal cell carcinoma and promotes Interleukin-6-induced apoptosis suppression and cell motility. Journal of Biological Chemistry 281, 7756–7764. 16428383 10.1074/jbc.M509668200

