# Supplementary material for: Lipidomic perspectives on the role of lactosylceramides in inflammation and disease: A narrative review
Source: Expert Rev Mol Med. 2026 May 22;28:e26. doi: 10.1017/erm.2026.10054 (PMC13312804; doi:10.1017/erm.2026.10054)
Supplement: Hicks and Whiley supplementary material [file S1462399426100544sup001.docx]

| System | Disease | Sample type | Key findings | Reference |
| --- | --- | --- | --- | --- |
| Immune | Paediatric bacterial vs viral infection | Plasma and serum | LacCer(d18:1/16:0) and (d18:1/24:1) increased in bacterial vs viral infection. | [54] |
|  | Pneumonia or upper respiratory tract infection (URI) | Serum | LacCer(d18:1/12:0) increased in pneumonia vs URI.  LacCer(d18:1/14:0) increased between acute and recovered pneumonia | [55] |
|  | COVID-19 | Serum and urine | Total LacCer decreased in non- COVID-19 infection vs COVID-19 and control.  LacCer(d18:1/16:0), (d18:1/22:0) and (d18:1/24:0) increases with COVID-19 severity. | [56] |
|  | Paediatric ulcerative colitis (UC)/ Crohn's disease (CD) | Serum | LacCer(d18:1/16:0) discriminated UC from CD. | [57] |
|  | Paediatric UC/ CD | Serum | LacCer(d18:1/16:0) discriminated UC from CD.  In UC LacCer(d18:1/16:0) correlates with CRP, WBC and platelet count. | [58] |
|  | Paediatric UC/ CD | Plasma and serum | LacCer(d18:1/16:0) increased in UC and CD compared to symptomatic controls. | [59] |
|  | UC | Serum and colon tissue | In serum total LacCer increased in UC vs control, whereas (d18:1/24:1) decreased in severe disease stage.  Increased LacCer(d18:1/16:0), (d18:1/24:0) in inflamed tissue vs control. | [60] |

**Supplementary Table S1. Summary of lipidomics studies reporting LacCer in the immune system**

| System | Disease | Sample type | Key findings | Reference |
| --- | --- | --- | --- | --- |
| Endocrine | Overweight | Serum | LacCer(d18:1/12:0) increased in overweight patients from baseline to 3-year follow-up period.  LacCer(d18:1/12:0) positively correlates with brachial–ankle pulse wave velocity (baPWV). | [37] |
|  | Metabolic syndrome (MetS) | Serum | LacCer(d18:1/14:0), (d18:1/16:0) and (d18:1/24:1) were decreased in MetS.  LacCer(d18:1/18:1) was positively associated with HOMA-IR.  Total LacCer was significantly associated with increased inflammatory markers (IL-6, CRP, fibrinogen and Intercellular Adhesion Molecule 1 (ICAM). | [33] |
|  | Obesity ± MetS | Plasma | No significant LacCer between groups | [38] |
|  | Impaired fasting glucose (IFG) | Serum | Positive correlation between LacCer(d18:1/12:0), fasting glucose levels and baPWV. | [39] |
|  | IFG | Plasma | LacCer(d18:1/16:0) decreased as fasting glucose and BMI increased. | [40] |
|  | Insulin resistance (IR) and type 2 diabetes (T2D) | Plasma  Serum | LacCer(d18:1/14:0), (d18:1/16:0) and (d18:1/24:1) were inversely associated with HOMA-IR and T2D. | [41] |
|  | IR in obesity ± T2D | Serum  Muscle biopsy | Serum LacCer(d18:1/14:0) positively associated with muscle TG accumulation in obese IR .  Serum LacCer(d18:1/22:0) predictive of whole-body IR. | [42] |
|  | Gestational diabetes with 8-year progression to T2D | Plasma | LacCer(d18:1/14:0) ,(d18:1/16:0), (d18:1/20:0), (d18:1/22:0), (d18:1/24:1) negatively associated with T2D disease risk. | [43] |
|  | T2D risk | Plasma | LacCer(d18:1/16:0) not associated with T2D disease risk | [44] |
|  | T2D risk | Plasma | Total LacCer negatively associated with T2D disease risk.  LacCer(d18:1/18:1), (d18:1/18:2) negatively associated with T2D disease risk.  Lower LacCer levels may underlie higher T2D risk in South Asians. | [28] |
|  | Obesity ± T2D | Adiposomes  Endothelial cells | LacCer(d18:1/16:0), (d18:1/18:0), (d18:1/20:0), (d18:2/24:1) increased in OB-T2D adiposomes.  Adiposome uptake and fusion with endothelial cells was 4-fold higher in OB-T2D vs control.  Increased LacCer(d18:1/16:0) and (d18:1/18:0) in the OB-T2D fusion cells. | [45] |

**Supplementary Table S2. Summary of lipidomics studies reporting LacCer in the endocrine system**

**Supplementary Table S2. (cont). Summary of lipidomics studies reporting LacCer in the endocrine system**

| System | Disease | Sample type | Key findings | Reference |
| --- | --- | --- | --- | --- |
| Endocrine | T2D | Plasma | Total LacCer was increased in T2D (drug naïve) compared to controls | [20] |
|  | Diabetic retinopathy | Eyes | Total LacCer is increased in diabetic vs control.  LacCer(d18:1/20:0) and (d18:1/26:0) increased in diabetics vs control. | [21] |
|  | T2D ± macroalbuminuria (MA) | Plasma | LacCer(d18:1/18:0), (d18:1/20:0), (d18:1/24:0), (d18:1/26:0) increased in T2D vs control.  LacCer(d18:1/18:0), (d18:1/20:0), (d18:1/24:0), (d18:1/26:0) decreased in T2D + MA vs T2D.  LacCer(d18:1/22:0) and (d18:1/24:1) increased in T2D + MA vs T2D  LDL associated LacCer(d18:1/26:0) decreased in T2D ± MA.  Total LacCer associated with HDL2 and HDL3 decreased in T2D.  LacCer(d18:1/16:0) associated with HDL2 and HDL3 were decreased in T2D ± MA.  Long-chain LacCer associated with HDL2 were decreased in T2D ± MA. | [46] |
|  | T2D +MA and chronic kidney disease (CKD) | Plasma lipoproteins | In LDL LacCer(d18:1/16:0),(d18:1/18:0), (d18:1/20:0), (d18:1/22:0), (d18:1/24:0), (d18:1/24:1) increased in CKD vs controls. | [47] |
|  | Obese IR ± non-alcoholic fatty liver disease (NAFLD) or ± non-alcoholic steatohepatitis (NASH) | Serum, liver and adipose tissue | In serum LacCer(d18:1/14:0) was positively associated with NAFLD+, whereas total LacCer was positively associated with NAFLD- .  In liver tissue total LacCer and LacCer(d18:1/24:1) increased in NASH +.  In visceral adipose tissue LacCer(d18:1/14:0), (d18:1/16:0), (d18:1/24:0) and (d18:1/24:1) had a positive association with adiponectin. | [19] |
|  | NAFLD | Serum | LacCer(d18:1/16:0) was associated with higher hepatic attenuation and inversely associated with T2D and fasting glucose levels after 5-year follow up period. | [48] |
|  | NAFLD | Serum and liver | LacCer(d18:1/24:1) and (d18:1/16:0) are associated with lower hepatic TG levels. | [49] |
|  | Mammographic breast density (MBD) | Fasting blood sample | LacCer(d18:1/14:0) positively associated with volumetric percent density (VPD). | [50] |
|  | MBD | Fasting blood sample | LacCer(d18:1/14:0) mediated the association of BMI at ages 10 and 18 with VPD. | [51] |
|  | Polycystic ovarian syndrome (PCOS) ± obesity ± IR | Serum | Decreased LacCer (d18:1/16:0) in obese PCOS participants ± IR. | [52] |

**Supplementary Table S3. Summary of lipidomics studies reporting LacCer in Cardiometabolic health**

| System | Disease | Sample type | Key findings | Reference |
| --- | --- | --- | --- | --- |
| Cardiovascular | Post-cardiac surgery | Plasma | LacCer(d18:1/24:1) increases throughout hospital stay.  LacCer(d18:1/24:0) lower than reference range throughout hospital stay. | [34] |
|  | Atherosclerosis | Aortic tissue | Total LacCer accumulates in atherosclerotic lesions. | [17] |
|  | Atherosclerosis | Carotid plaque | Increased total LacCer in plaque of symptomatic vs asymptomatic.  Total LacCer positively correlates with IL-6, MCP-1 and MIP-1β. | [32] |
|  | Atherosclerosis | Aortic intima and media tissue | Increased LacCer(d18:1/16:0), (d18:1/22:0), (d18:1/22:1), (d18:1/24:0) in intima plaque, whereas LacCer(d18:1/18:0), (d18:1/18:1) decreased in intima plaque compared to unaffected intima tissue.  LacCer(d18:1/16:1), (d18:1/17:0), (d18:1/18:2) and (d18:1/20:1) absent in intima plaque compared to unaffected intima tissue. | [35] |
|  | Atherosclerosis in SLE | Plasma | Total plaque area (TPA) negatively correlates with LacCer(d18:1/16:0), (d18:1/20:0), (d18:1/22:0), (d18:1/24:0), (d18:1/24:1) and total LacCer at baseline.  At 1-year follow-up TPA positively correlates with LacCer(d18:1/16:0), (d18:1/24:1) and total LacCer. | [18] |
|  | Atherosclerosis in SLE | Plasma | LacCer(d18:1/18:1), (d18:1/24:1), (d18:1/26:0) and (d18:1/26:1) differentiate SLE + atherosclerosis to SLE patients.  LacCer(d18:1/18:0) and (d18:1/20:0) differentiate white vs African American SLE with and without atherosclerosis. | [113] |
|  | Acute myocardial infarction (AMI) | Monocytes | Increased LacCer(d34:1) in AMI compared to control. | [114] |
|  | Paediatric cholelithiasis | Serum | Decreased LacCer(d18:1/24:0) and (d18:1/24:1) and cholelithiasis, whereas LacCer(d18:1/16:0) and (d18:1/18:1) were increased. | [36] |

**Supplementary Table S4. Summary of lipidomics studies reporting LacCer in the central nervous system**

| System | Disease | Sample type | Key findings | Reference |
| --- | --- | --- | --- | --- |
| Nervous | Multiple sclerosis- relapsing remitting multiple sclerosis (RRMS) or progressive multiple sclerosis (PMS) | Serum | LacCer(d18:1/16:0) and (d18:1/16:1) increased in PMS vs control.  LacCer(d18:1/20:1) decreased in both RRMS and PMS vs control.  LacCer(d18:1/22:0) increased in both RRMS and PMS vs control.  LacCer(d18:1/24:1) increased in PMS vs control. | [68] |
|  | Mild cognitive impairment (MCI) | Plasma | LacCer(d18:1/24:1)  associated with MCI. | [69] |
|  | Alzheimer’s disease (AD) | Serum | LacCer(d18:1/12:0) associated with increased risk of all cause dementia and Alzheimer’s. | [70] |
|  | AD | Neuroglioma cells with H4APPsw mutation | Total LacCer 2-fold increase in APPsw cells compared to wild-type. | [24] |
|  | Idiopathic Parkinson's disease (IPD), dementia with Lewy bodies (DLB), multiple system atrophy (MSA), AD and progressive supranuclear palsy (PSP) | Plasma | Total LacCer increased in all group’s vs control. LacCer(d18:1/14:0) and (d18:1/16:0) increased in IPD, DLB, AD, PSP and MSA; LacCer(d18:1/16:1) increased in IPD, DLB, PSP and MSA; LacCer (d18:1/18:0) increased in DLB, AD and MSA; LacCer (d18:1/18:1) increased in DLB; LacCer (d18:1/20:1) increased in DLB and AD; LacCer (d18:1/22:1) increased in DLB and AD; LacCer (d18:1/22:2) increased in DLB and AD; LacCer (d18:1/24:0) decreased in MSA; LacCer(d18:1/24:1) and (d18:1/24:2) increased in DLB. | [25] |
|  | GBA1-associated Parkinson's disease (*GBA1*-PD) | Plasma, PBMCs, and CSF | Plasma: LacCer(d18:1/18:0), (d18:1/22:0) and (d18:1/22:1) increased in *GBA1*-PD, whereas LacCer(d18:1/20:0) was decreased.  CSF: LacCer(d18:1/18:0) and (d18:1/20:0) was decreased. | [71] |
|  | Parkinsons disease cognitive impairment | Plasma | Total LacCer increased in all PD cognition groups vs controls. | [115] |

**Supplementary Table S5. Summary of lipidomics studies reporting LacCer in the renal system**

| System | Disease | Sample type | Key findings | Reference |
| --- | --- | --- | --- | --- |
| Renal | Type 1 diabetes mellitus + MA ± chronic kidney disease (CKD) | Plasma | LacCer(d18:1/14:0), (d18:1/16:0), (d18:1/18:0), (d18:1/24:0), (d18:1/24:1), (d18:1/26:0) and (d18:1/26:1) decreased in MA vs control  Increased LacCer(d18:1/22:1) in MA vs control  LacCer(d18:1/14:0), (d18:1/16:0), (d18:1/18:0), (d18:1/24:0), (d18:1/26:0) and (d18:1/26:1) decreased in CKD vs control  Increased LacCer(d18:1/22:1) in CKD vs control  Decreased LacCer(d18:1/14:0), (d18:1/16:0), (d18:1/18:0), (d18:1/24:0), (d18:1/26:0) and (d18:1/26:1) in MA + CKD vs control  Increased LacCer( d18:1/22:1) in MA + CKD vs control | [111] |
|  | T2D +MA and chronic kidney disease (CKD) | Plasma lipoproteins | In LDL LacCer(d18:1/16:0),(d18:1/18:0), (d18:1/20:0), (d18:1/22:0), (d18:1/24:0), (d18:1/24:1) increased in CKD vs controls. | [47] |
|  | Paediatric CKD | Serum | LacCer(d18:1/16:0) and (d18:1/24:0) increased in CKD vs control. | [72] |
|  | Systemic lupus erythematosus (SLE) ± nephritis | Plasma and urine | Plasma: No significant differences.  Urine: LacCer(d18:1/16:0) increased in nephritis. | [73] |
|  | Polycystic kidney disease (PKD) | Kidney tissue | LacCer(d18:1/16:0), (d18:1/18:0) and (d18:1/18:1) increased in PKD.  LacCer(d18:1/19:0) and (d18:1/20:0) absent in PKD.  LacCer(d18:1/24:0) and (d18:1/24:1) decreased in PKD. | [112] |
|  | Lupus nephritis (LN) | Serum and urine | Serum: LacCer(d18:1/22:0) and (d18:1/24:0) increased in LN vs controls, with no sex differences observed.  Urine: LacCer(d18:1/16:0), (d18:1/22:0), (d18:1/24:0) and (d18:1/24:1) and total LacCer increased in LN vs controls.  LacCer(d18:1/16:0), (d18:1/24:0) and (d18:1/24:1) twofold higher in LN males than in LN females. | [22] |

**Supplementary Table S6. Summary of lipidomics studies reporting LacCer across biological systems**

| System | Disease | Sample type | Key findings | Reference |
| --- | --- | --- | --- | --- |
| Digestive | Paediatric cholelithiasis | Serum | Decreased LacCer(d18:1/24:0) and (d18:1/24:1) and cholelithiasis, whereas LacCer(d18:1/16:0) and (d18:1/18:1) were increased. | [36] |
| Integumentary | Burn injury | Skin | Total LacCer, LacCer(d18:1/22:1) and (d18:1/24:0) increased in burn injury tissue vs control. | [23] |
|  | Paediatric non-severe burn injury | Plasma | LacCer(d18:1/24:0) and (d18:1/26:0) discriminated paediatric non-severe burn injury vs controls. | [53] |
| Cancer | Colorectal cancer (CC) | Colon tissue | LacCer(d18:1/16:0), (d18:1/18:0), (d18:1/22:0), (d18:1/24:0) and (d18:1/24:1) increased in CC tissue vs control. | [61] |
|  | CC and advanced adenoma (AA) | Faecal | LacCer(d18:1/16:0) increased in CC vs AA | [62] |
|  | Anaplastic astrocytoma | Serum | LacCer(d18:1/12:0) increased in Anaplastic astrocytoma vs control | [63] |
|  | Cholangiocarcinoma (CCA) | CCA tumour and liver tissue | LacCer(d18:1/16:0), (d18:1/h16:0) and (d18:1/h24:0) increased in CCA tissue. | [64] |
|  | Hepatocellular carcinoma (HCC) | Plasma | LacCer(40:3) increased in HCC vs controls. | [65] |
|  | Prostate cancer | Urine exosomes | LacCer(d18:1/16:0) increased in prostate cancer vs control. | [66] |
|  | Melanoma metastasis | Plasma | Total LacCer decreased in metastasis vs control. | [27] |
|  | Multiple myeloma (MM) and asymptomatic monoclonal gammopathy (MGUS) | Bone marrow plasma | Decreased total LacCer in MM vs MGUS. | [67] |
| Other | Biological aging | Plasma | Total LacCer decreased with 0.25–1.00 year per each SD concentration increase. | [26] |
|  | Niemann-Pick C disease (NPC) | Plasma | Total LacCer was decreased in NPC vs controls. | [76] |
